# A critical appraisal of advances in integrated CO_2_ capture and electrochemical conversion†

**DOI:** 10.1039/d4sc06642a

**Published:** 2025-01-24

**Authors:** Ahmed Badreldin, Ying Li

**Affiliations:** a J. Mike Walker '66 Department of Mechanical Engineering, Texas A&M University College Station TX 77843 USA yingli@tamu.edu; b Artie McFerrin Department of Chemical Engineering, Texas A&M University College Station TX 77843 USA

## Abstract

This perspective work examines the current advancements in integrated CO_2_ capture and electrochemical conversion technologies, comparing the emerging methods of (1) electrochemical reactive capture (eRCC) though amine- and (bi)carbonate-mediated processes and (2) direct (flue gas) adsorptive capture and conversion (ACC) with the conventional approach of sequential carbon capture and conversion (SCCC). We initially identified and discussed a range of cell-level technological bottlenecks inherent to eRCC and ACC including, but not limited to, mass transport limitations of reactive species, limitation of dimerization, impurity effects, inadequate *in situ* generation of CO_2_ to sustain industrially relevant current densities, and catalyst instabilities with respect to some eRCC electrolytes, amongst others. We followed this with stepwise perspectives on whether these are considered intrinsic challenges of the technologies – otherwise recommendations were disclosed where appropriate. Furthermore, technoeconomic analysis (TEA) was conducted using a net present value (NPV) model to determine the minimum selling prices (MSPs) for CO, HCOOH, CH_3_OH, C_2_H_5_OH, and C_2_H_4_ as target products based on cell-performance metrics from contemporary literature for SCCC, eRCC, and ACC. Additionally, sensitivity analyses were performed, focusing on cell-level parameters (voltage requirements, Faradaic efficiencies, current density), production scale factors, and other relevant variables (levelized costs of electricity and stack). This analysis sheds light on the cost-driving factors influencing commercial viability, revealing key techno-economic challenges for eRCC, particularly with liquid products. However, it also identifies optimization opportunities in current designs. By pinpointing critical areas for improvement, this work helps advance electrochemical CO_2_ reduction technologies towards more sustainable and economically competitive applications at different scales.

## Abstract 2

This perspective critiques advancements in integrated CO_2_ capture and electrochemical conversion, contrasting emerging methods like eRCC *via* amine or (bi)carbonate pathways and direct ACC with traditional sequential capture and conversion strategies.

## Graphical abstract

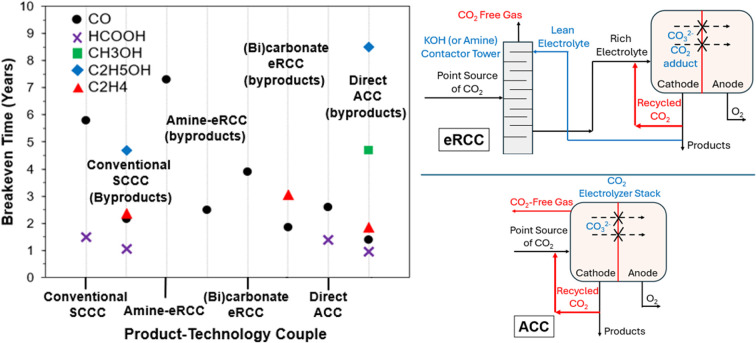

## 1 Introduction

Over the past century, global energy consumption increased tenfold, aligning with a more than threefold rise in the global population, with various forecasts indicating ongoing growth in both energy requirements and population in the upcoming decades.^1^ Consequently, it is anticipated that various industries, extending beyond the green energy sector, will face heightened demand. This projected surge in demand underscores the significance of implementing sustainable and energy-efficient production processes. As per the Intergovernmental Panel on Climate Change (IPCC) agreement, there is a non-binding mandate to achieve a reduction in global CO_2_ emissions to 32 Gt (7.6% annual reduction) from 2020 to 2030 in order to meet the 1.5 °C temperature increase target.^2^ Although a notable decline in global emissions was registered in 2020, predominantly due to industrial slowdowns from the COVID-19 pandemic, an alarming rebound of 4.8% to approximately 34.9 Gt of CO_2_ has been recorded since.^3^ To that end, a plethora of approaches have been, and continue to be, examined towards curbing emissions and achieving the carbon-neutrality target. Seeing as a large portion of anthropogenic carbon emissions is from the industrial sector, it is logical to see contemporary efforts being directed towards complementing, or replacing altogether, environmentally malignant methods of producing chemical feedstocks such as hydrogen (H_2_), carbon monoxide (CO), ethylene (C_2_H_4_), and ethanol (C_2_H_5_OH).^4–7^ For example, H_2_ is a pivotal feedstock in the petrochemical industry for an array of secondary products, such as ammonia (NH_3_), gas-to-liquid (GTL), and feed chemicals (*i.e.*, methanol), to name a few.^8–11^ To date, over 95% of global H_2_ is considered gray H_2_, whereby it is sourced from steam methane reforming (SMR).^12^ However, green H_2_ production from electrochemical water splitting powered by renewable electricity has seen an exponential increase in advancement over the past decade, in terms of electrocatalyst design,^13–15^ electrolyzer design,^16–18^ and meaningful process integration.^19,20^ Emerging technologies have been capitalizing on the expected shift from the conventional hydrocarbon-based heating to cleaner high-temperature H_2_ combustion and reduction, as is the case with the direct reduction of iron for the steel industry.^21^ Similarly, an explosion of interest has been directed towards CO_2_ electrolysis, promising to deliver the same essential petrochemical feedstocks but through renewable electricity powered electrochemical CO_2_ reduction reaction (eCO_2_RR).^22^ Electrochemical approaches not only have the intrinsic advantage of achieving the same desired product in an environmentally benign manner, but also provide a pathway for utilizing intermittent renewable energy, namely solar and wind energy, which expedites the complementary adoption of greener technologies. By 2030, it is projected that 3.5 Gt of CO_2_ emissions from conventional pathways of chemical production will be decoupled through eCO_2_RR. Projections estimate the need to source upwards of 18 PW h (petawatt hour) of renewable electricity to achieve this.^23^ This highlights the apparent interdependence between the needed innovation and delivering capacity of the renewable energy sector and the sought after success of eCO_2_RR in achieving a positive and substantial environmental footprint.

To date, great progress has been made in eCO_2_RR with respect to achieving industrially relevant current densities, energy and carbon efficiency, stability, and Faradaic efficiency. This, however, is predominantly for C_1_ carbon products with room for improvement for C_2_ and C_2+_ hydrocarbons (*i.e.*, C_2_H_4_) and oxygenates (*i.e.*, C_2_H_5_OH).^24,25^ Based on contemporary eCO_2_RR results, several technoeconomic analyses (TEA) studies were undertaken with a degree of conservatism in terms of performance metrics to compare the current economic value proposition of eCO_2_RR with conventional methods.^26,27^ For instance, Gao *et al.* recently reported that eCO_2_RR produced C_2_H_4_ and C_2_H_5_OH are not yet competitive with conventional production methods. Therein, CO and formic acid (HCOOH) attained levelized eCO_2_RR production costs at approximately 0.45 and 0.47 USD per kg, less than 2023 market prices of 0.6 and 0.68 USD per kg, respectively.^28^ Sensitivity analyses have pointed to improvements in Faradaic efficiency having the most significant effect on levelized production costs of C_2_ products, especially C_2_H_4_ and C_2_H_5_OH.^29^ This is primarily due to alleviation of costly downstream separation costs when target product selectivity is subpar. Cathodic activity enhancements and lower levelized costs of renewable electricity will also lower the levelized cost of C_2_ and C_2+_ products from eCO_2_RR routes. The aforementioned TEA, and most others, rely on the rather customary sequential carbon capture and storage (CCS) followed by eCO_2_RR. This is mainly due to the established infrastructure and high technology readiness levels (TRLs) of amine-based adsorbents for high-volume and high-concentration CO_2_ point-source industrial emissions. This is a far cry from emerging adsorbents such as, but not limited to, ionic liquids and covalent organic frameworks (COFs) that act as capture agents in integrated electrochemical CO_2_ conversion systems.^30–32^ Notwithstanding, the conventional CCS route followed by eCO_2_RR seemingly has intrinsic challenges and limitations, namely a penalizing energy-demanding regeneration step, as well as storage and transport logistics and costs of captured CO_2_.^33^

The conventional sequential CO_2_ capture and conversion (SCCC) technology approach is generally regarded as the most mature technology in eCO_2_RR, primarily because researchers have historically tested electrocatalytic performance using pure CO_2_ feed streams and have benefited from advancements in gas diffusion electrode (GDE) technologies. Carbon capture (CC) technologies themselves are at a relatively high TRL, with several commercially viable technologies already in operation (TRL 9) such as post-combustion amine capture and pre-combustion natural gas processing.^34^ Therefore, the generally lower TRL for SCCC is primarily limited by the secondary electrochemical conversion step, rather than the initial carbon capture process. This limitation is largely due to challenges related to non-ideal selectivity and stability, issues that will be explored in greater detail in this work. Therefore, while SCCC stands out as the most developed approach, only a few of its target products have reached TRLs that are promising enough for foreseeable commercial realization. These products are primarily carbon monoxide and formic acid, and their eCO_2_RR has been recently ranked at TRLs of 5–6 and 3–5, respectively.^35^ Most other SCCC targeted products (*e.g.*, ethylene, acetic acid, *etc.*) rank at a TRL of 4 or lower, including tandem electrochemical CO_2_ to CO followed by CO to C_2_H_4_, which has been ranked at a TRL of 4.^35^ For the recently emergent integrated routes of CO_2_ capture and conversion introduced and discussed in this perspective, they are generally categorized to be between TRLs of 3–4. Their generally lower TRL is primarily based on selectivity, scalability, and stability challenges that have not yet been fully addressed at larger scales of operation.

### 1.1 High-level shortcomings of sequential CO_2_ capture and conversion (SCCC)

The assumption that CO_2_ is as abundant and accessible as water in the context of CO_2_ electrolysis is often incorrect. Unlike water electrolysis, CO_2_ electrolysis facilities need a constant, uninterrupted feed of highly pure CO_2_ to match the desired production capacity and account for inefficiencies. The required flow rate of highly pure CO_2_ is critical to ensuring stable and efficient operation. This overlooked point is essential for the successful implementation of CO_2_ electrolysis technology. To that end, selecting an appropriate CO_2_ transport method is crucial, considering capacities, geographical location, security, and distance requirements between the capture point and utilization facilities. Upstream carbon capture is thought to be economically meaningful at a relatively high minimum threshold of annual CO_2_ capture – at least 0.1 million metric tons (MMT) of CO_2_ per year – which may not always be in an acceptable geographic fit with a renewable energy source capacity to meet electrolysis requirements. Currently, CO_2_ is transported by trucks, trains, ships, and pipelines. Although specific pressure vessels employed on trucks and trains are suitable for transporting small quantities – approximately 1–5 MMT per year – over short distances (a few hundred kilometers), the economic and safety feasibility favor pipelines for large quantities (∼100 MMT per year) and longer distances. Globally, a mere 6500 kilometers of CO_2_ pipelines exist, primarily in the USA and Canada, albeit mainly for enhanced oil recovery (EOR) efforts. Typical onshore transport costs range from 1–11 USD per ton per 100 mile, while offshore costs range from 0.05–0.53 USD per ton per 62.1 miles depending on the technology and transport capacity – transportation cost and transportation capacity are inversely proportional.^36,37^ Therefore, the benefit of decentralization which is conventionally associated with electrochemical processes in general is not very straightforward and requires an abundance of planning for at-scale commercially relevant CO_2_ electrolysis initiatives, specifically when it comes to the conventional sequential CCS-eCO_2_RR framework. To that end, as it appears, centralized facilities are required for large capacity CO_2_ capture (>0.1 MMT of CO_2_ per year), subsequent storage, and eventual utilization through conversion to value-added products. The needed volume of CO_2_ influx can potentially be met from several neighboring point-source emissions forming a cluster such as those found in petrochemical complexes. Notwithstanding, in an effort to circumvent the holistic sizing bottlenecks of the sequential CCS-eCO_2_RR framework, an exciting approach entails integrating CO_2_ capture and conversion in a single coupled system or device, dubbed electrochemical reactive capture of CO_2_ (eRCC).^38^

Another facet which is worth posing is the realism undertaken during TEA – at least for this stage of this contribution focusing on the non-electrochemical aspects of CCS-eCO_2_RR. For instance, the value proposition of environmentally conscious electrochemical technologies depends on using renewable energy, yet TEA reports often assume 8000 operational hours per year for amortization.^29^ This estimate is a far cry from the more realistic 2000 hours per year typically associated with onshore renewable energy, varying by generation type, such as wind or solar PV, and geographic location.^39^ If factored in, this in turn would be reflected as even higher capital expenditure (CAPEX) costs and thereby higher levelized production costs for target products. Furthermore, renewable energy capacities employed in TEA base assumptions tend to be open ended in the sense that availability is not an issue, which again is not necessarily the case for typical production capacities at least greater than 10 MW. Moreover, and much like water electrolyzers which have a higher TRL than CO_2_ electrolyzers, a primary system-level issue facing electrochemical stacks connected solely to renewable energy sources is the intrinsic dynamic operation behavior due to the intermittency of said renewable energy sources.^40^ Differentiation in the applied voltage, due to power availability, does not guarantee the same operational stability tested under the conventional lab-scale chronoamperometric (CA) or chronopotentiometric (CP) behavior towards sustained current density performance. This is another example of unrealistic steady-state assumptions used in relevant TEAs. In fairness, some efforts by the Janáky group showcased that power ON–OFF scenarios over a course of a week on a zero-gap CO_2_ electrolyzer to CO do not significantly affect performance. However, the testing duration of the study is too short to be conclusive and applicable to the myriad of catalyst-product couples.^41^

In an effort to sustain target production rates with an intermittent renewable power source, Esposito and Fthenakis introduced a TEA model that optimizes current density profiles for dynamically operated electrolyzers – albeit water electrolyzers.^42^ It is worth noting that under the dynamic nature of renewable energy accelerated degradation may occur on any of the cell components which could significantly increase the replacement rate of the stack – from once every 7 years in current TEAs to the order of months. Although some efforts and deliberation have been made pertaining to a trade-in scheme with tapping into grid electricity during periods of low renewable energy and providing surplus renewable energy during periods of higher supply, this scheme would need further life cycle assessment (LCA) studies to prove maintenance of carbon neutrality efficacy with the assumptions undertaken in the literature today. It is important to consider such aspects since unstable production costs over the operational lifetime for a CO_2_RR product that exhibited economic feasibility under static conditions, irrespective of the technology or product, can be problematic for investors and deployment of CO_2_ electrolysis if not addressed early on during low TRL development efforts. Notwithstanding, other approaches that tap into advancing technologies such as decoupling anodic and cathodic reactions through redox mediators or through direct storage of renewable energy *via* solar redox-flow batteries (RBF) and integrated solar flow batteries (SFB) are currently being investigated for their upscaling potential and suitability for coupling with solar-fuel production.^43–45^

An alternative approach adopted by a few research groups to tackle the intermittency issue focuses on redesigning entire electrolyzers, rather than solely optimizing electrocatalysts. This strategy aims to maximize production rates, reduce voltage requirements, and maintain high selectivity for the target product, ultimately improving cathodic efficiency and the overall energy efficiency of the system. Briefly, the standard thermodynamic potential (*E*°) for CO_2_ reduction reactions is directly proportional to energy efficiency because a higher *E*° indicates a lower theoretical energy requirement relative to the actual cell potential, provided the Faradaic efficiency remains high. Additionally, energy efficiency increases with higher Faradaic efficiency and lower applied potential, as both reduce energy losses and improve the effective utilization of the input energy. Typically, advanced electrolyzers primarily tackle this through minimizing kinetic limitations of the CO_2_ electrolyzers – in turn achieving maximized current densities (*J*_max_) at lower applied potential bias. Briefly, aqueous flow-by (AFB) cells (standardly known as flow-cells) typically achieve a stable *J*_max_ of 0.5 A cm^−2^,^46^ followed by GDE flow-cells with neutral catholyte (GDEN; *J*_max_ ∼0.6 A cm^−2^), membrane-electrode assembly (MEA) cells with humidified CO_2_ inlets and anion exchange membrane (GDEM; *J*_max_ ∼1 A cm^−2^),^47^ a GDEA (*J*_max_ ∼1.4 A cm^−2^) cell which employs a CO_2_ inlet and alkaline catholyte,^48^ and finally a novel flow-through induced dynamic triple-phase boundary (TPB) cell dubbed the FTDT cell (*J*_max_ ∼3.37 A cm^−2^).^49^ Wen *et al.* recently compared their FTDT cell with the counterpart flow-cells and MEA cells to conclude that gas–electrolyte–catalyst interfaces, local electrode microenvironment, and a tenfold decrease in diffusion-layer thickness (*δ*_DL_) contribute to the concurrent CO_2_, electrons, protons, and product transfer and thus facilitate current densities >3 A cm^−2^.^49^ Energy efficiency values of 60, 40, and approximately 28% are registered for 0.01, 1, and 3 A cm^−2^ current density, translating to less renewable energy requirements to meet the same CO_2_RR product production capacity – albeit their work was targeting CO production and not higher value-added chemicals.

### 1.2 Approaches to integrated CO_2_ capture and conversion

**Fig. 1:**
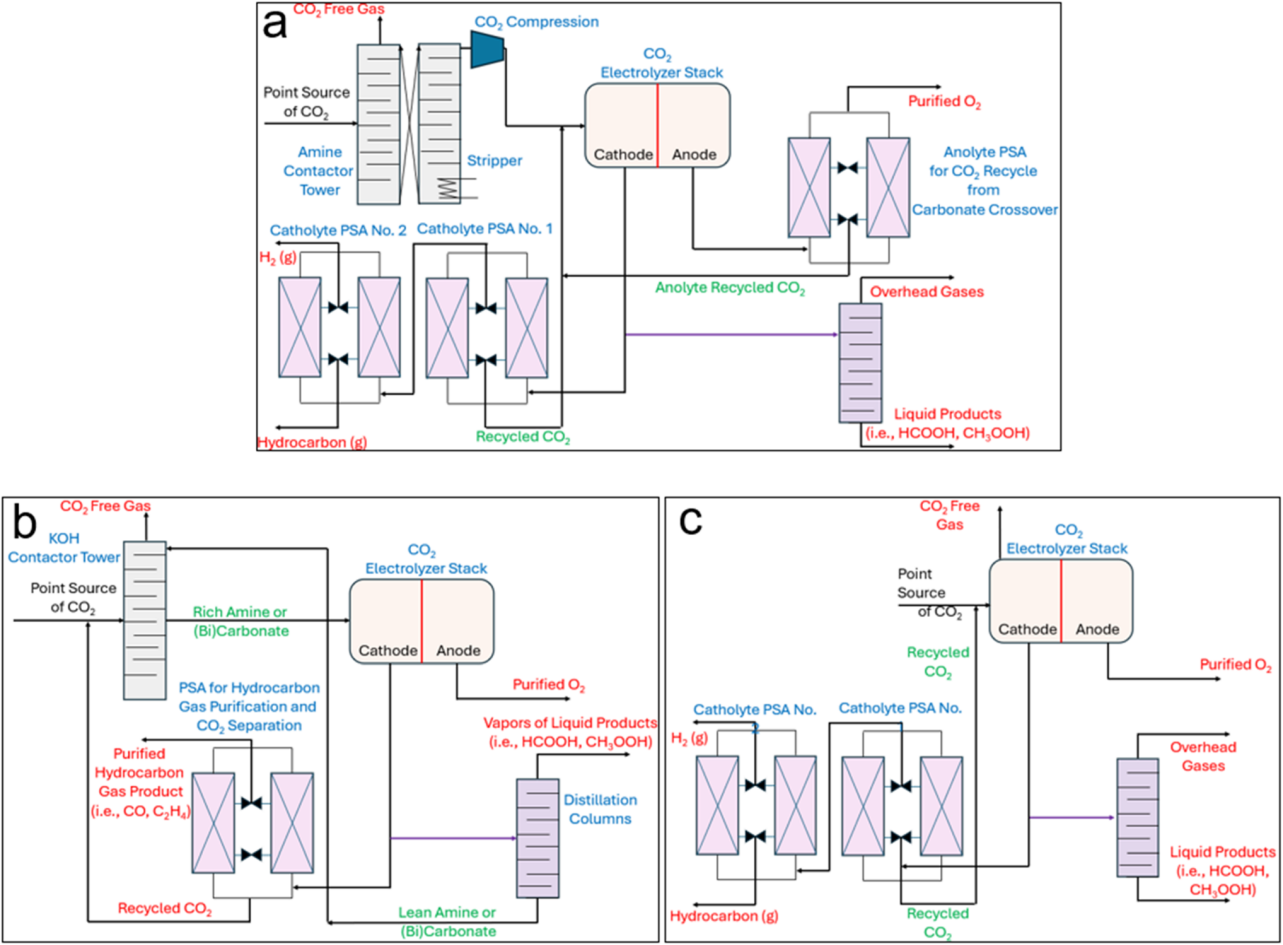
(a) Process flow diagrams (PFDs) of the conventional sequential CO_2_ capture, stripping, compression, and electrochemical reduction route (System 1), (b) amine- and (bi)carbonate-mediated eRCC approaches, and (c) direct adsorptive capture and conversion (ACC).

In an effort to address many of the previous points, integrated CO_2_ capture-eCO_2_RR, or eRCC, captains an array of sub-pathways that aim towards the same overarching goals: shortcutting the standalone step of separating and purifying CO_2_ from a mixed gas feed stream. Under the governance of the conventional CCS-eCO_2_RR (Fig. 1a), CO_2_ emissions from a point-source first pass through an amine contactor tower which selectively strips the CO_2_ from other gases. Following this, the rich amine stream enters a stripper which simultaneously regenerates the amine adsorbent and desorbs the CO_2_ in a purified form. In this context, the conventional amine scrubbing process costs 50–150 USD per ton of captured CO_2_ – over 60% of the cost is attributed to the regeneration and compression steps of CO_2_.^50^ It goes to show that qualitatively large portions of the associated costs can be curbed through appropriate integrated pathways. Compression of the pure CO_2_ stream should only serve to push the CO_2_ through the needed piping and not necessarily to have it enter the CO_2_ electrolyzer under high pressure. In the conventional AEM type electrolyzer used for eCO_2_RR, an appreciable amount of CO_2_ is directly converted to carbonate which, due to the anion exchange membrane, is allowed to pass to the anode. There, local acidic conditions from the anodic water oxidation reaction cause the carbonate to become CO_2_ again. Therefore, a pressure-swing-absorber (PSA) is installed at the outlet of the anode to separate anodic O_2_ from the crossed-over CO_2_. The CO_2_ can be recycled back as part of the cathode feed. Similarly, downstream of the cathodic effluent, at least one PSA is needed – although typically two are used. This is because the unreacted CO_2_, varying amounts of byproduct H_2_, and eCO_2_RR product gases (CO, C_2_H_4_) need to be sequentially separated and purified to meet market qualities. In the case where a target liquid product is generated at the cathode (*i.e.*, CH_3_OH, HCOOH, C_2_H_5_OH), then a distillation column is needed to further concentrate and separate the product from the electrolyte and mixed unreacted CO_2_. The unreacted CO_2_ and electrolyte can either be flashed first before being recycled or recycled directly as catholyte depending on the process-specific composition of the stream.

eRCC technologies including, but not limited to, amine- and (bi)carbonate-mediated routes circumvent the initial upstream CO_2_ capture and stripping steps in Fig. 1a by offering a single solution to act as both a capture agent and electrolyte for electroreduction (Fig. 1b). Therefore, the stripping and compression steps of Fig. 1a are eliminated. Instead, in eRCC routes the raw point-source CO_2_ saturates the capture solution upstream in a contactor before feeding the CO_2_-rich capture solution to the electrolyzer. Therein, the same product separation constraints that govern the conventional system persist except for a key advantage and holistic disadvantage. Under eRCC routes, the electrolyzer design and/or capture solution prevent CO_2_ to cross over to the anolyte. This eliminates the need for a PSA at the effluent of the anode, which decreases costs. However, since the capture solution/electrolyte is a liquid, liquid products need to be separated from it through distillation. The expectedly high volume of capture electrolyte that would need to pass through distillation dictates sizing the column accordingly – both for the CAPEX and operating expenditure (OPEX). This can be expected to significantly add to the final production cost of liquid products through eRCC routes. Targeted gas products would not require a distillation step and instead the capture electrolyte would simply be regenerated through recirculating it back to the contactor to become saturated with CO_2_. Moreover, an emerging solid-based process will be disclosed and examined in this work which entails a single hybrid GDE that targets selective on-stream pre-concentration of CO_2_ before reaching the catalytic interface for reduction (Fig. 1c). In this route, regeneration is not needed assuming that the hybrid GDE is durable for its operational lifetime.

### 1.3 Outline of the perspective

In this perspective, a preview of recent advances and the current state of capture media CO_2_ electrolysis and solid-state enhanced conversion techniques will be presented and critiqued. Key technological and economic bottlenecks will be identified for several technology-product couples to draw out key areas of opportunity for development. Briefly, this work is structured to initially give a guiding summary and overarching perspective of the technological landscape of conventional eCO_2_RR (*i.e.*, SCCC), with a dedicated focus on integrated CO_2_ capture and electrochemical conversion approaches. These primarily encompass amine-mediated (Section 2) and (bi)carbonate-mediated eRCC (Section 3) approaches, highlighting their motivations, limitations, and bottlenecks, along with perspectives on overcoming these challenges. Additionally, we incorporate the adsorptive capture and conversion (ACC) route into the broader discussion and dedicate Section 4 to exploring hybrid MOF-modified GDEs and mixed matrix membranes (MMM) for dilute CO_2_ gas electrolysis. Contemporary literature benchmarks for key technology-product couples will be identified, discussed in detail, and contrasted where appropriate with other couples of the same product type. For reference, a tabulation of said benchmarks is disclosed in Table S1† and is based on electrochemical performance metrics (*i.e.*, required voltage, achievable current density, Faradaic efficiency, *etc.*). Further, through a conservative TEA (Section 5), an NPV model is used to estimate the minimum selling price (MSP) of five key products – namely CO, HCOOH, CH_3_OH, C_2_H_4_, and C_2_H_5_OH – using the predefined contemporary literature benchmarks in Table S1.† We will conclude with Section 6 by reiterating and building key perspective points that are both aimed at the cell- and system-level for the discussed integrated CO_2_ capture and electrochemical conversion approaches.

## 2 eRCC through amine-mediated approaches

### 2.1 Mechanistic obscurity limits allowable product spectrum

Electrochemical RCC has gained traction because it holistically circumvents sizing bottlenecks, energy requirements of regenerating CO_2_ capture media, and compression costs of purified CO_2_ within the sequential CCS-eCO_2_RR framework. Herein, the capture medium is both electrochemically stable and conductive to act as the electrolyte during eRCC. Since amines form ionic species upon chemisorption with CO_2_, namely ammonium carbamate ([NH_4_][H_2_NCO_2_]) and ammonium bicarbonate ([NH_4_][HCO_3_]), a conductive electrolyte is attained, although steric effects of the target amine and bulk viscosity effects need to be accounted for to prevent transport limitations and excessive Ohmic losses. Specifically, it is the N–C bond of carbamates and their carboxylic acids that offer tunable reactivity since the amine–CO_2_ adduct is by virtue irreversible (−65 to −90 kJ mol_CO_2__^−1^).^51^ Unlike conventional eCO_2_RR in aqueous bicarbonate electrolyte, amine eRCC seems to offer an advantage of potentially lower downstream separation costs since unreacted CO_2_ remains within the capture media and would not escape with gaseous products.

While the integration of amine-mediated eRCC systems offers intriguing potential, their performance metrics often fall short of conventional eCO_2_RR setups, with lower product selectivities, commercially irrelevant current densities, and limited operational stability. In contrast, recent advancements in conventional SCCC pathways, such as the work by Fang *et al.*,^52^ demonstrate remarkable efficiency, achieving 100% CO Faradaic efficiency at an ultra-high current density of 1200 mA cm^−2^ in an alkaline catholyte using innovative cobalt-porphyrin-lined mercurated graphyne blocks combined with N-doped graphene (Hg-CoTPP/NG) cathode design. Contrastingly, the Sargent group reported the highest reported performance for amine–CO_2_ conversion to CO with 72% FE at 50 mA cm^−2^ using a silver (Ag) sputtered cathode and a 30% (w/w) monoethanolamine (MEA) electrolyte.^53^ Interestingly, the initial FE towards CO was less than 5%, with the balance being the conventionally parasitic hydrogen evolution reaction (HER). The initial poor performance was attributed to the electric double layer (EDL) on the cathode which allowed ammonium counterions to electrostatically prevent carbamate ions (MEACOO^−^), assumed to be the reactant in this study, from reaching the buried catalytic interface. Reducing the EDL thickness through introducing low hydration radius alkali cations, namely Cs^+^, lowers the transport limitations of the reactive carbamate anion and allows FE toward CO to increase from 5 to 72% at 50 mA cm^−2^. Notwithstanding, the benchmark cell performance for SCCC^52^ results in a 33 times higher reaction rate toward CO production compared with the amine-mediated eRCC^53^ benchmark when factoring for current density and corresponding FE. Targeting another prominent C_1_ product, namely formate (HCOO^−^), Chen *et al.*^54^ employed a bismuth (Bi) bulk metal electrode towards amine-mediated eRCC which exhibited a 60.8% FE towards the target product.^54^ This was achieved upon dosing the electrolyte with 0.1 w/w% cetyltrimethylammonium bromide (CTAB) surfactant to suppress the HER, albeit with an inadvertent 40% drop in activity to 10 mA cm^−2^. Contrary to the design rationale by the Sargent group,^53^ the reported performance by Chen *et al.* was not in fact attributed to MEA-CO_2_ carbamate adducts, but to the direct reduction of dissolved CO_2_ remaining as the active species, whereby carbamate and ethanolammonium (MEAH^+^) simply offer supporting electrolyte properties. Similar notions were supported by dedicated studies, notable of which is that by Leverick *et al.* who investigated active species across different amines and control electrolytes (*i.e.* KHCO_3_, KCl) on silver electrodes, and found no direct correlation between the carbamate concentration and resultant FE of produced CO.^55^ However, due to the reported differences in performance between different amine eRCC agents it is argued that carbamate may act as a sink for CO_2_ due to the aqueous-CO_2_ equilibrium, wherein COO^−^ is released upon depletion of feed HCO_3_^−^ and dissolved CO_2_.

**Fig. 2:**
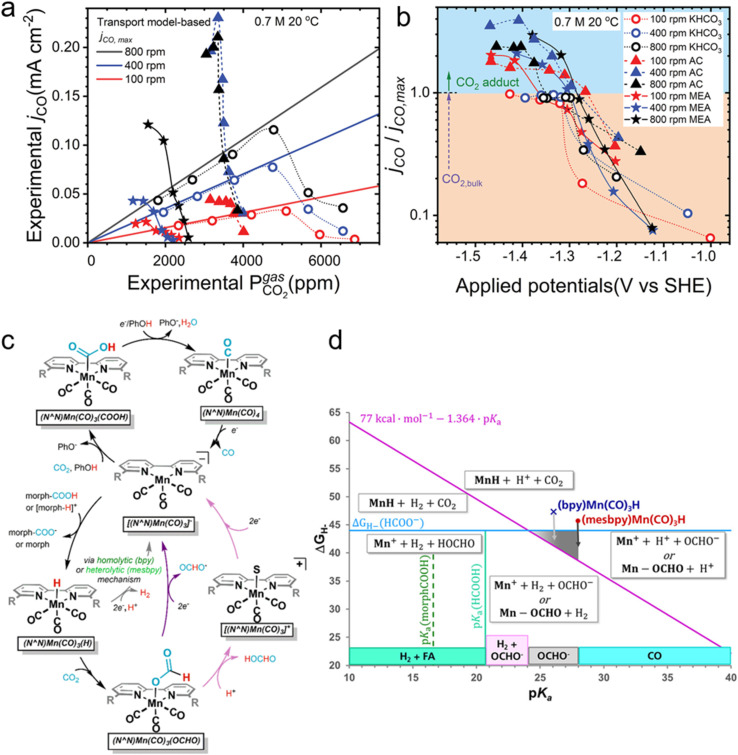
Experimental and transport model-based maximum partial current density for the reduction of dissolved CO_2_ in capture solution at equilibrium with the partial pressure of CO_2_ determined using a gas chromatogram. Each experiment is carried out by increasing the current and determining the potential needed to drive total current densities of 1, 4, 8, 12, 20, and 28 mA cm^−2^. (a) Experimental partial current for CO in 0.7 M solutions of potassium bicarbonate, ammonium carbamate (AC), and CO_2_-loaded MEA. (b) Ratio between partial current density for CO and the model-based maximum CO partial current if the CO_2_ source is purely dissolved CO_2_ in the bulk of the electrolyte in the 0.7 M solutions in (a). Reprinted with permission from ref. 56. Copyright 2023, Cell Press. (c) Proposed mechanism of the electrocatalytic reduction of CO_2_ to give CO (top) or FA (bottom). Direct protonation of Mn(OCHO) is indicated by light purple arrows, and reduction followed by formate loss is indicated by dark purple arrows. H_2_ production is indicated by gray arrows, and the stoichiometry shown is for the heterolytic pathway. (d) Plot of hydricity *versus* p*K*_a_ in acetonitrile. Solid lines represent boundaries for speciation (boxed). Formate is only obtained in the gray triangle. For FA, formate, and H_2_, the hydricity or apparent hydricity must be sufficient enough to obtain the products. Reprinted with permission from ref. 58. Copyright 2020, American Chemical Society.

As a seeming compromise between the two mechanistic viewpoints, Shen *et al.* recently performed a combined grand canonical density functional theory (GCDFT) with electrochemical characterization and revealed that unbound dissolved CO_2_ is the primary carbon species being consumed during amine-mediated eRCC on silver electrodes.^56^ Briefly, using a gas-tight rotating cell electrode (RCE) to study the gas–liquid and liquid–solid interfacial properties in reduction of CO_2_-rich MEA electrolyte, it was found that at high overpotentials the maximum allowable partial current density for CO generation is not solely dependent on the overhead partial pressure of CO_2_ in the cell, suggesting involvement of the CO_2_–amine adduct as a secondary carbon source only at high overpotentials (Fig. 2a and b). The transport model-based maximum allowable current density for CO generation (*J*_CO,max_ = 2 × FE_CO_ ×*k*_m,CO_2__ × *C*_CO_2_,bulk_) is simply a function of the FE towards CO (FE_CO_), the bulk concentration of dissolved CO_2_ (*C*_CO_2_,bulk_), and the film mass transfer coefficient of CO_2_ in the electrolyte (*k*_m,CO_2__), whereby the latter is a function of the hydrodynamics in the proximity of the liquid–catalyst interface.^57^ Nevertheless, the ratio of experimental partial current density for CO relative to the theoretical maximum remained largely unaffected by the rotation speed of the electrode. This indicates that the enhancement is proportional to the mass transfer coefficient at the liquid–catalyst interface. Further, a direct correlation between carbamate concentration and partial current density of CO was confirmed. This supports the possibility of direct carbamate reduction at more negative potentials as proposed by theoretical GCDFT models undertaken in the work.

Due to the array of possible amines, their binding affinities to CO_2_, reactivity of the resultant adducts (if they are in fact the reactants), steric effects, and p*K*_a_ ranges, it is envisaged that performance can be improved compared to the above-mentioned contemporary results. Following on this, Bhattacharya *et al.* utilized morpholine as the amine eRCC agent, generating a mixture of both carbamate and carbamic acid, over different Mn-based molecular catalysts known for their intrinsically high CO selectivity in the presence of weak acids (*i.e.*, phenol).^58^ It was found that CO remains as the predominant product in the absence of morpholine; however, a complete product distribution switch favoring H_2_ occurs upon the introduction of morpholine to the weakly acidic system as shown from the mechanistic pathways in Fig. 2c. In contrast, the sole presence of morpholine switches the products to both H_2_ and formic acid, with negligible CO. This was largely attributed to the resultant electrolytic p*K*_a_, as well as the metal hydride [M–H]^(*n*−1)+^ cleavage mode within the different electrolytic systems. There are briefly three modes of metal hydride cleavage, namely homolytic or heterolytic dissociation to produce atomic H or H^+^ cations, respectively, or the hydride donor ability (hydricity, 
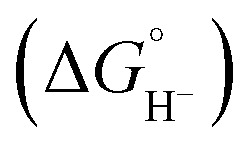
).^59^ The latter is described as the heterolytic bond dissociation free energy of the M–H to yield the parent metal complex ([M]^+^) and the hydride (H^−^). Briefly, in the disclosed work by Bhattacharya *et al.*, H_2_ is favored under fast kinetics corresponding to homolytic H_2_ production, whereas when the electrolytic system solely allows heterolytic H_2_ generation then formic acid is dominant.^58^ The above-mentioned relationship between p*K*_a_ and hydricity is shown in Fig. 2d.

### 2.2 Bottlenecks of amine-mediated eRCC

Interestingly, the overwhelming majority of amine-mediated eRCC work tends to produce CO, formate, and parasitic H_2_, with no reports of C_2+_ hydrocarbons or oxygenates that we were able to identify in relevant literature. From conventional eCO_2_RR, it is known that the binding strength of the catalytic surface to the intermediate adsorbed (*i.e.*, *CO, where (*) represents binding on an active site) influences product selectivity.^60^ The adsorbate-surface binding energetics (*i.e.*, ΔG_*CO_) must be balanced with the overpotential requirements of the HER (*η*_H_2__) on the same catalytic surface to prevent a negative limiting potential between eCO_2_RR and HER (favoring hydrogen production). Bulk surfaces such as tin (Sn) and Bi which exhibit a high *η*_H_2__ and positive, or weak, ΔG_*CO_ tend to produce formate, while those with moderate *η*_H_2__ and ΔG_*CO_ tend to form CO (Ag and Au) or hydrocarbons (Cu-based).^61,62^ Notwithstanding, in the case with amine-based eRCC the use of certain transition metal based catalysts is prohibitive due to the chemical instability. This is particularly notable for Cu, which was found to suffer from severe corrosion in an aqueous MEA electrolyte, showcased by atomic absorption microscopy (AAS) and scanning electrochemical microscopy (SECM).^63^ This comes as no surprise since amines are employed as non-ammoniacal etching etchants for Cu printed circuit boards.^64^

As was mentioned, a key benefit of eRCC is removing upstream amine regeneration costs and downstream separation costs of crossover CO_2_. Assuming an amine-based eRCC system and a conventional eCO_2_RR system operate at the same cell performance in terms of activities, voltage requirements, and selectivities, then the cost analysis will naturally favor amine-based eRCC. However, the seemingly intrinsic limitation of amine-mediated eRCC of solely producing CO and formate is worrisome since both these products are already electrochemically produced commercially with competitive market prices using conventional eCO_2_RR. Adding to this, the apparent commercially irrelevant current densities and selectivities of amine-based eRCC suggest that cell-level catalyst and electrolytic bottlenecks will hold back cell performance comparison for quite some time – before a fair technoeconomic (TEA) comparison with conventional eCO_2_RR can be trusted. Further, although there is value for sodium or potassium formate, acetate, and lactate salts, it is their carboxylic acid counterparts that have a greater market value. The needed ion exchange resins for converting formate salts into their carboxylic acids for example will add additional Ohmic losses, which would increase the required potential bias and consequently production cost.

Although amine-mediated eRCC promises an opportunity for controllable product selectivity, a few cell- and systems-level limitations seem to intrinsically hinder this approach's potential. Firstly, compared with conventional alkaline electrolytes, the mass transport limitations of CO_2_ in amine solutions are considerably higher. As a simple exercise, viscosity of 30 w/w% MEA, a primary amine, is approximately 3.5 mPa s,^65^ relative to about 1.0 and 1.2 mPa s for 0.1 M KHCO_3_ and 1.0 M KOH, respectively, used in conventional eCO_2_RR.^66^ The inverse relationship between viscosity and diffusion coefficient in the Stokes–Einstein relationship showcases notable transport limitations in the MEA system. Furthermore, irrespective of the amine type used, viscosity is shown to increase monotonously with increasing CO_2_. That means under the typical feed CO_2_ (100 mol%) used in the literature for amine-mediated eRCC, the diffusivity of CO_2_ can be expected to be limiting compared to that in aqueous electrolytes. Moreover, although several groups found that secondary, tertiary, and quaternary amines may aid in improving binding affinities with reaction intermediates, and therefore eCO_2_RR selectivities, the steric hindrances and increased hydrogen bonding of higher order amines correspondingly increase their respective solution viscosity. High viscosities during electrochemical reactions result in a higher Ohmic loss, which increases the required voltage to achieve a given current density. Since the viscosity of amine-based solutions is known to decrease exponentially with increasing temperature, it would be interesting to see the trade-off effect temperature can have between existing transport limitations and captured CO_2_ levels for amine-mediated eRCC that seemingly hold current densities below 100 mA cm^−2^.

Secondly, C–C dimerization is limited in the presence of amine-based electrolytes. This is not just because Cu-based catalysts cannot be utilized due to their poor stability in amine electrolytes. Other multi-functional catalysts including doped carbons, Ag and Au-based, early-transition metal-based, Fe/Co/Ni-based, and In/Sn-based catalysts have been demonstrated to attain C_2+_ products, although generally at lower efficiencies compared to Cu-based catalysts.^67^ For example, Wu *et al.* synthesized N-doped graphene quantum dots (NGQD) and showed that at applied potentials more negative than −0.61 V (*vs.* RHE) effective C–C dimerization occurs, predominantly producing C_2_ and C_3_ products.^68^ The highest FE for C_2+_ hydrocarbon and oxygenate products (C_2_H_4_, C_2_H_5_OH, CH_3_COO^−^, and 1-propanol) surpassed 55% at −0.75 V (*vs.* RHE), with a 90% FE towards eCO_2_RR. It is noteworthy that partial current densities for C_2_H_4_ (∼50 mA cm^−2^ at −1.03 V (*vs.* RHE)) and C_2_H_5_OH (∼25 mA cm^−2^ at −0.74 V (*vs.* RHE)) are comparable to those of commercial Cu nanoparticles under similar testing conditions. Conventionally formate-forming metals like Sn have also been utilized towards electrochemical C–C coupling. Highly faceted and crystalline SnO_2_ particles have been shown to attain FE towards C_2_H_5_OH up to 10%, along with trace amounts of C_2_H_4_, CH_3_COOH, and 1-propanol, whereby it was concluded that catalyst–electrolyte interactions determine early-stage C–C coupling of C_1_ species towards multi-carbon products.^69^ The plethora of C–C coupling mechanisms proposed and demonstrated to date in aqueous media are typically dependent on binding affinities between the catalytic surface and different reaction intermediates (*i.e.*, *CO, *CHO, *COH, *CH_2_, *CH_3_, and *HCHO), applied potentials, and pH that thermodynamically sway both the activity and selectivity trends.^67^ The lack of C_2+_ products reported in amine-mediated eRCC suggests that there is an underlying mechanistic limitation towards C–C dimerization in the presence of amines as reactive capture agents. We postulate that these may be a result of: (i) viscosity and transport limitations limiting the diffusion of reactants and intermediates needed during C–C coupling, (ii) strong hydrogen bonding between amines, H_2_O, and CO_2_ derived species creates solvation shells that stabilize intermediates making them less reactive towards dimerization, (iii) high proton availability due to amine–water interactions may favor the more kinetically facile HER, and (iv) regardless of whether carbamates are the feed reactants or a supporting electrolyte, their thermodynamic stability lowers kinetically meaningful C–C coupling from their CO_2_ adducts.

The premise of RCC in general, and eRCC in specific, targets point-source CO_2_ effluents whereby gas compositions are typically in the range of 5–20% CO_2_,^70,71^ with potentially an array of feed gas impurities including N_2_, O_2_, SO_*x*_, and NO_*x*_.^72^ It has been found that both O_2_ and NOx (as low as 0.83%) can cause complete deterioration of the progression of the CO_2_RR by promoting the parasitic oxygen reduction reaction (ORR) and nitrogen reduction reaction (NRR).^73,74^ Additionally, trace metal impurities (*i.e.*, Fe^2+^, Zn^2+^) in the electrolyte can be easily reduced and deposited on the catalyst surface, deactivating the catalyst.^75^ Although strategies have been introduced to deal with these impurity effects in conventional eCO_2_RR, to the best of our knowledge, there has not been a study on gas impurity effects on amine-mediated eRCC. Besides that, we have not found a single report using dilute CO_2_ feeds (CO_2_ in N_2_ or Ar balance) in amine-mediated eRCC; this is surprising as the overarching goal of eRCC is to directly utilize flue gas CO_2_.

Another challenge that seems to be typically overlooked in transport limiting amine-mediated eRCC is the electrolyzer design. Briefly, flow-cell configurations have been widely adopted in electrochemical applications including eCO_2_RR,^76^ water electrolysis,^8^ and others due to their lower Ohmic losses which thereby enhances activity.^77^ For gas phase eCO_2_RR, GDEs can typically deliver current densities >200 mA cm^−2^ without notable Ohmic losses. Briefly, GDEs consist of a catalyst layer, a microporous layer, and a macroporous layer which promotes mass transport of feed gas. In flow-cell configurations, feed CO_2_ passes from the macroporous layer inwards towards the catalyst layer in gaseous form. The key which makes flow-cells superior to H-cells for eCO_2_RR is that the local CO_2_ environment around the catalyst has 4 orders of magnitude higher gas diffusion coefficients (0.15 cm^2^ s^−1^) than in aqueous solutions (1.92 × 10^−5^ cm^2^ s^−1^) due to the tri-phase boundary (TPB).^78^ Since there are very few dedicated reports on TPB effects in amine-mediated eRCC, it is difficult to ascertain why activities herein are still limited well below 100 mA cm^−2^. In amine-mediated eRCC, flow-cells have been employed for longer term stability tests; however, the activity is still found to be limiting. Therefore, this results in stability tests that monitor irrelevant current densities when compared to the status quo of conventional eCO_2_RR. We believe that this is primarily a result of the lower diffusion coefficients of the CO_2_–amine adduct or the low concentration of dissolved CO_2_ around the catalytic surface microenvironment. Unlike conventional eCO_2_RR flow-cells where the feed gas reached the TPB as gaseous CO_2_, in amine-mediated eRCC the reactive species are fed with the electrolyte, which is the capture agent solution, and thus, the overall transport of the reactive species to the GDE is hindered. This mass-transport limitation is largely overlooked in eRCC in general and especially for amine-mediated eRCC, which poses challenges in comparing with conventional eCO_2_RR systems in TEA models for decision makers.

## 3 eRCC through (bi)carbonate-mediated approaches

### 3.1 Origin and guiding principles of (bi)carbonate-mediated eRCC

To address the costly issue of carbonate formation noted in conventional systems (Fig. 1a), eRCC through (bi)carbonate-mediation ensures minimal to no feed CO_2_ loss within the electrolyzer. Neutral or alkaline pH CO_2_RR suffers from low CO_2_ utilization efficiencies and the single pass conversion efficiency (SPCE) is quite low (<25% when targeting C_2+_ products in alkaline environments), leading to high regeneration costs.^79,80^ Since separation of CO_2_ is an energy-intensive process (2–4.4 GJ per ton of CO_2_),^81^ unreacted CO_2_ inadvertently increases the overall energy requirements of the process substantially. Therefore, with a focus on eliminating downstream CO_2_ separation at the anodic effluent, the overall technoeconomic comparison between (bi)carbonate-mediated eRCC and conventional eCO_2_RR becomes holistically desirable – providing similar cell performance and target reaction products can be achieved. Further, a system that efficiently utilizes bicarbonate (HCO_3_^−^) as a carbon source is highly compelling, namely due to the much higher CO_2_ concentration sequestered in a saturated aqueous solution of KHCO_3_ (∼3.3 M CO_2_) compared to a mere 33 mM for saturated CO_2_ in water. Another intrinsic advantage of the direct reduction of bicarbonate is circumventing the local acidification problem at the cathode during conventional gas-fed CO_2_ electrolysis. Briefly, during (bi)carbonate eRCC the local drop in pH from protons released during water dissociation goes towards releasing CO_2_ from (bi)carbonate, as opposed to getting consumed in the HER. To that end, the Berlinguette group were the first to successfully showcase non-formate product generation from (bi)carbonate eRCC –wherein formate production from (bi)carbonate reduction was demonstrated in 1983 by Hori but at less than 1 mA cm^−2^ current density.^82,83^ The work demonstrated the direct reduction of 3.0 M solution of bicarbonate, yielding a CO FE of 81 and 37% at 25 and 100 mA cm^−2^, respectively, which is comparable to that achieved when the electrolyte is saturated with gaseous CO_2_.^82^ In their design, a bipolar membrane (BPM) is employed, as opposed to a conventional anion exchange membrane (AEM), such that water dissociation occurring under reverse-bias provides protons to the catholyte which oxidizes HCO_3_^−^ to CO_2_ for reduction at the catalyst interface. Following this, a capture unit was formulated and integrated with the eRCC bicarbonate BPM electrolyzer, whereby CO_2_ is captured by passing it over a high surface area manifold containing a liquid KOH sorbent, generating HCO_3_^−^.^84^ Upon acidic oxidation of the HCO_3_^−^ anion from BPM generated protons, OH^−^ (KOH) is recycled back to the capture unit in a closed loop setup. The resultant energy efficiency of this system was reportedly 37% higher than an approach where a standard gas-fed CO_2_ electrolyzer is used.^85^ This approach became quite popular for other direct reduction of carbonate work that targeted gaseous CO, as well as C_2_H_4_ and C_2_H_5_OH which have been achieved through eRCC of carbonate but to a much smaller extent.

A key advantage of (bi)carbonate eRCC is the opportunity to tap into a plethora of know-how from the more mature water electrolysis field. For example, unlike gas-fed CO_2_ electrolysis approaches, herein a metallic cathode can be used directly without the need for an aerophilic carbon support layer. Metal foams have been used directly both in the catalyst design stage and in stability testing of substrate supported membrane configurations in zero-gap flow cells used in water electrolysis. The high specific surface area, permeability, hydrophilicity, and reproducibility of metal foams provide sufficient transport of aqueous bicarbonate feedstock through the electrode. For example, Zhang *et al.* demonstrated utilization of such porous metallic electrodes in a simplified assembly which exhibited high CO_2_RR selectivity, namely 95 and ∼55% FE towards CO at 4 and 1 atm of feed capture agent pressure, respectively, at 100 mA cm^−2^.^86^ Although many investigations attribute maximized CO_2_RR activity to surface area, given the same intrinsic catalytically active site, mass transport is typically overlooked. To illustrate the effect of this in a bicarbonate eRCC, Kim *et al.* conducted tests on a variety of electrospun carbon nanofibers of different diameters and sizes, all coated with Ag nanoparticles.^87^ The results showed that electrodes with greater permeability enhanced *in situ* generation of CO_2_ from HCO_3_^−^ oxidation by facilitating more efficient transport of HCO_3_^−^ ions from the flow plate to the catalytic interface. However, these highly permeable electrodes exhibited lower CO_2_ utilization due to the reduced surface areas. As a result, the most effective electrodes tested featured an intermediate fiber size whereby a tradeoff between high surface area for CO_2_ utilization and permeability (fiber size dependence) for overcoming transport limitations is reached. This offers a perspective as to why under bicarbonate eRCC operation, the porous Ag-metal foams showed almost double the selectivity towards CO compared to a standard Ag-based composite carbon cathode.^86^

Irrespective of the target electrochemical reaction product, in (bi)carbonate-mediated eRCC the reaction proceeds through 4 key cathodic reactions and a conventional OER anodic reaction under the constraints of a reverse-bias BPM configuration. For example, in the case of CH_4_ production (an 8-electron transfer process) four cathodic electrochemical reactions involve the *in situ* generation of CO_2_ from bicarbonate through acidification [8HCO_3_^−^ (aq.) + 8H^+^ (aq.) → 8CO_2_ (aq.) + 8K^+^ (aq.) + 8H_2_O (l)], where the H^+^ is provided from reverse-bias water dissociation within the BPM. The *in situ* released aqueous CO_2_ can then participate in the standard 8 electron-transfer reduction forming CH_4_ and stoichiometrically generating an 8 to 1 mole ratio of KOH to feed CO_2_. To prevent acidification of CO_2_, acid–base neutralization with the present KOH occurs which regenerates the initial bicarbonate carbon capture moiety [7CO_2_ (aq.) + 7KOH (aq.) → 7 KHCO_3_ (aq.)]. Carbonate (K_2_CO_3_ (aq.)) formation then occurs through base chemistry between KHCO_3_ (aq.) and KOH (aq.). CH_4_ production is favored in local acidic microenvironments on Cu, as opposed to C_2+_ products favoring local alkalinity which stabilizes dimerization – unless under low coordination Cu number.^88^ Lees *et al.* utilized the intrinsic advantages of the *in situ* generation of CO_2_ at the cathode and the local acidity from H^+^ influx by the BPM within a 3.0 M KHCO_3_ electrolyte to generate CH_4_ at an unprecedented yield (molar ratio of produced CH_4_ to unreacted CO_2_ gas) of 34% at 120 mA cm^−2^, compared to 3% for the previous benchmark in conventional eCO_2_RR.^89^ Further, by utilizing a cationic surfactant (CTAB) at ∼3 mM concentrations, FE towards CH_4_ increased from 0 to 27% at 400 mA cm^−2^. For reference, a benchmark in CH_4_ production from conventional eCO_2_RR is reported to achieve a current density of 220 mA cm^−2^ with 62% CH_4_ FE using 4 V cell potential. This translates to an energy efficiency of 16% and consumption of 5 MJ per mol CH_4_, compared to ∼4% and 20 MJ per mol CH_4_, respectively, for the (bi)carbonate-mediated eRCC system by Lees *et al.*, which required 7.2 V to achieve 400 mA cm^−2^.^88,89^ Unfortunately, no stability tests were performed for the carbonate-mediated system, unlike the demonstrated 110 hours for the conventional eCO_2_RR methanation benchmark.^88^

Formate, as another C_1_ target product, was also achieved through the (bi)carbonate-mediated eRCC approach by several groups. Benchmarking catalytic performance under this approach is quite similar between two groups that directly reduced 3.0 M KHCO_3_ to formate. Briefly, Li *et al.* utilized Bi-nanoparticles on porous carbon and achieved 62 and 27% FE for formate at 100 and 400 mA cm^−2^, respectively,^90^ and Gutiérrez-Sánchez *et al.* demonstrated 58 and 38% FE for formate at 100 and 400 mA cm^−2^, respectively,^91^ using Sn/SnO_2_ on porous carbon supports. Interestingly, both reported results showcased approximately 4 and 7 V cell potential to achieve 100 and 400 mA cm^−2^ current density, respectively, under similar BPM type electrolyzers. It was found that controlling the feed flowrate has important effects on the resultant FE and corresponding energy efficiency (EE). For example, in the work of Gutiérrez-Sánchez *et al.* an optimal EE of 27% can be achieved using 50 mA cm^−2^ with an electrolyte flowrate of 5 mL cm^−2^. Correspondingly, high current densities (>300 mA cm^−2^) led to lower EE (∼10%) using lower feed flowrates (0.5 mL cm^−2^) but at high production of formate (>40 g L^−1^).^91^ Contrastingly, Wang *et al.* used conventional eCO_2_RR to achieve partial formate current densities up to 450 mA cm^−2^ at ∼2.2 V cell voltage, FE to formate up to 97%, and developed a clever porous solid electrolyte (PSE) interposer with low flowrate carrier N_2_ gas that transports HCOOH vapor that is condensed to purified (∼100 wt%) HCOOH solution.^92^ This performance was in part attributed to a novel, highly electroactive, selective, and stable grain-boundary enriched Bi-based catalyst. Based on this, the disparity between conventional and (bi)carbonate enabled eRCC towards formate is quite evident from an energy point of view – approximately 3 times the energy is needed to achieve the same partial current density through the (bi)carbonate eRCC approach. Again, it is believed that this energy requirement disparity could be shrunk through utilizing state-of-the-art BPM which have lower Ohmic losses and energy requirements for water dissociation. Further, although technoeconomic analyses (TEA) tend to be undertaken between conventional eCO_2_RR and emerging eRCC approaches, the stability factor – typically overlooked even for conventional eCO_2_RR – is almost completely ignored in eRCC approaches. Most eRCC investigations are limited to system level optimizations of design and operation aspects to achieve the highest SPCE, EE, and FE towards the target product, with stability effects being left out of scope.

As an unfortunate running theme thus far, most targeted products generated through eRCC approaches seem to be limited to C_1_ products. As noted previously, amine-mediated eRCC has intrinsic limitations in generating C_2+_ products due to the instability of Cu-based electrocatalysts in said amine capture electrolyte. However, the case for (bi)carbonate-based eRCC is slightly different. The governing premise of (bi)carbonate eRCC revolves around *in situ* generated CO_2_ from acid–base chemistry between HCO_3_^−^ and protons supplied by the CEM side of the BPM. The separation distance, or interposer thickness, between the CEM and the cathodic electrocatalyst surface was found to be important towards the production of C_2+_ products. This point was first noticed by the Sargent group, whereby using Cu-based catalysts known to favor dimerization in conventional eCO_2_RR, the total FE of C_2_ products (C_2_H_4_ and C_2_H_5_OH) remained below 14%.^93^ The initial design used exhibited a spacing of about 60 μm which corresponded to a volume fraction of *in situ* gaseous CO_2_ at the catalyst layer being in the order of 2 vol% – too low for adequate dimerization to occur. Therefore, the total FE towards C_2_ products is much lower than what would be expected under conventional eCO_2_RR. For reference, an array of Cu-based catalysts have achieved greater than 60% ethylene FE at high current densities (>200 mA cm^−2^) and prolonged operational stabilities (>50 hours) in neutral and alkaline eCO_2_RR within both flow cells and MEAs.^48,94–97^

### 3.2 Contemporary advances in (bi)carbonate eRCC

Modeling of chemical species under (bi)carbonate-mediated eRCC was then performed by Lee *et al.* to ascertain that a threshold interposer distance between the CEM and catalyst layer needs to be met to allow the local pH to sufficiently drop allowing for *in situ* CO_2_ generation.^98^ Kinetics calculations revealed that the CO_2_ concentration around the catalytic surface needs to be at least 4 vol% for dimerization to occur at higher current densities (>100 mA cm^−2^). Briefly, at commercially relevant current densities of 200–350 mA cm^−2^, the bulk pH at the interposer is only around 10 due to neutralization of carbonate and hydroxide anions to influxes of protons from the BPM.^99^ Interestingly, for dimerization to occur effectively at the catalytic surface, a locally high pH is preferred which exists naturally during eCO_2_RR around the cathode due to generation of OH^−^ (aq.).^100^ Therefore, to satisfy both criteria for C_2_ production through (bi)carbonate-mediated eRCC, larger interposer thicknesses (130–270 μm) were investigated and >4 vol% *in situ* CO_2_ reaches the catalytic surface for 200–350 mA cm^−2^ target current densities. In utilizing the aforementioned interposer thickness range with a Cu/CoPc-CNT cathode, Lee *et al.* demonstrated 47% FE for C_2+_ products at 300 mA cm^−2^ with a full cell voltage of 4.1 V with a resultant 56 wt% of the output gas being C_2_H_4_, and with CO, CH_4_, and CO_2_ combined accounting for less than 0.9 wt% – effectively the new benchmark in C_2_H_4_ production from (bi)carbonate-mediated eRCC.^98^ Achieving high product selectivity through this approach eliminates the energy demand for regenerating lost CO_2_, typically 95% lost to carbonate and unreacted form in conventional alkaline eCO_2_RR to C_2+_ products. This regeneration/separation energy cost herein is estimated to be in the order of 310 GJ per ton, approximately seven times the lower heating value of ethylene. It is worth noting that although performance was stable for the first 10 hours at 200 mA cm^−2^, what followed was a steady decline that was attributed to the high pH instability of the mixed cellulose ester (MCE) interposer used.

Other approaches towards achieving higher FE of C_2_ products within (bi)carbonate-mediated eRCC were demonstrated by Lee *et al.* whereby the local high pH around the cathodic active site of C_1_ to C_2_ conversion was maintained through the use of a bilayer tandem catalyst and bi-ionomers.^101^ Briefly, a tandem Cu–Ag catalyst was employed, through which the Ag catalyst was deposited atop Cu using a cation exchange ionomer. This approach allows the proton generated from the CEM layer of the BPM to be transported more favorably closer to the Ag catalyst in order to generate *in situ* CO_2_ from bicarbonate content. *In situ* generated CO on Ag can then travel through the GDE to the Cu catalyst to be reduced to C_2_H_4_ under a local alkaline microenvironment due to cation exchange ionomer trapping hydroxide anions. In doing so and incorporating a microporous hydrophobic PTFE layer into the Ag layer to suppress water transport for the HER, C_2+_ FE registered a maximum of 41.6% at 100 mA cm^−2^, with almost an equimolar amount of C_2_ hydrocarbon and oxygenates.

**Fig. 3:**
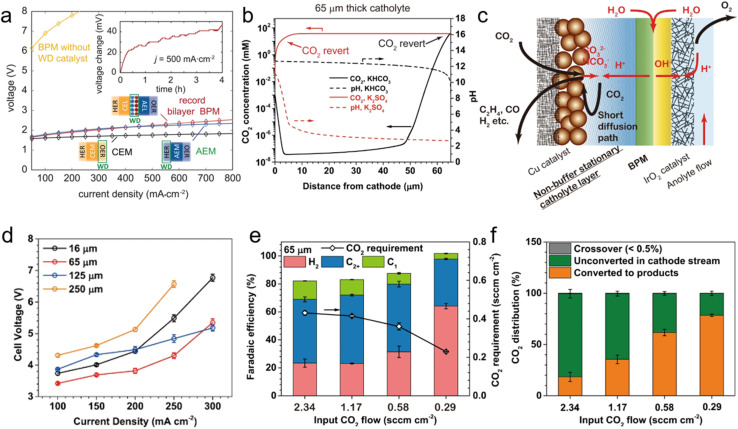
(a) Alkaline, acidic, and BPM water electrolyzers. BPM electrolyzers require >6 V to drive electrolysis at 50 mA cm^−2^ without additional WD catalysts (yellow). When the new bilayer WD catalysts are added, the performance substantially improves and is similar to that of reference AEM electrolyzers. Reprinted with permission from ref. 102. Copyright 2020, The American Association for the Advancement of Science (AAAS). (b) The CO_2_ (solid lines) and pH distributions (dashed lines) in the 65 μm-thick stationary catholyte (SC) layer. The positions where the (bi)carbonates revert to CO_2_ are marked (red for non-buffering and black for buffering electrolyte). (c) The schemes and the mass transfer in the SC BP membrane electrode assembly (SC-BPMEA). (d) The dependence of cell voltages on current density with different thickness of SC-BPMEA (35 °C with a CO_2_ flow rate of 10 sccm cm^−2^, a catholyte of 0.5 M K_2_SO_4_, and an anolyte of 0.1 M KHCO_3_). (e) FE distributions and the CO_2_ requirements (total CO_2_ converted to products) of the SC-BPMEAs at 65 μm thickness and input CO_2_ flow rates (sccm normalized by electrode area). (f) Carbon balance in SC-BPMEA with 65 μm 0.5 M K_2_SO_4_ at different input CO_2_ flow rates. Measurements in (e and f) were conducted at 35 °C and 200 mA cm^−2^, and the data were collected after 2 h of continuous operation. Reprinted with permission from ref. 104. Copyright 2022, Nature Publishing Group.

A key constraint that seems to limit the more widespread adoption of this approach is seemingly the voltage requirements needed to achieve meaningful current density (>200 mA cm^−2^) and at larger than 20% SPCE. This is because, as described earlier, the four reactions that progress on the cathode side are primarily reliant on proton provision from the BPM to oxidize HCO_3_^−^ into aqueous CO_2_. To that end, different electrolyzer configurations have been investigated towards lowering the energetic requirements of direct bi(carbonate) eRCC. BPMs typically require a large overpotential to achieve water dissociation at meaningful current densities. This can be seen in Fig. 3a whereby in the absence of a water dissociation catalyst in commercial BPMs, high voltage requirements are needed compared to commercial PEM and AEM membranes and their corresponding electrolyzers.^102^ Therefore, as an example, one of the lowest voltage requirements reported for bicarbonate-mediated eRCC using a BPM for cell operation greater than 200 mA cm^−2^ and 20% CO_2_ utilization was approximately 6 V. Notwithstanding, Zhang *et al.* showcased a 40% CO_2_ utilization efficiency at 2.3 V towards achieving a partial current density of KHCO_3_ to CO at 220 mA cm^−2^ using two primary modifications to the ‘conventional’ carbonate/bicarbonate-mediated eRCC cells.^103^ Namely, the authors employed a cationic exchange membrane (CEM; 50 μm Nafion) and replaced the anodic OER with a hydrogen oxidation reaction (HOR). CEMs are known for having higher ionic conductivity (mobility of H^+^ ∼36 × 10^−8^ m^2^ s^−1^ V^−1^) compared to AEMs whereby carbonate would have an ionic mobility in the range of 7.5 × 10^−8^ m^2^ s^−1^ V^−1^. The HOR is known to have overpotentials that are lower than the OER at the same current density. However, during the HOR the source of H_2_ needs to be considered due to levelized costs and carbon footprint considerations, wherein the latter could counteract and contradict the purpose of eCO_2_RR altogether.

It is worth noting that synergistically asynchronous advances are expected between different fields, even within electrochemistry. For example, and to the best of our knowledge, contemporary water dissociation catalysts (WDCs) developed towards reverse-bias water dissociation have only been reported once in conventional neutral electrolyte eRCC through the collaborative effort between Boettcher, Sinton, and Sargent.^104^ Therein, a stationary, non-buffering catholyte with an optimally designed catholyte layer thickness (Fig. 3c) effectively overcomes mass-transfer limitations while maintaining the necessary high local cathode pH (Fig. 3b) to favor C_2_ products. In a non-buffering catholyte, BPM-generated protons migrate further compared to a buffering catholyte, where protons are consumed at a faster rate. The optimal thickness of the SC-BPMEA was found to be around 65 μm (Fig. 3d), whereby larger thickness adds Ohmic losses and transport limitations, and thinner layers suffer from porosity control. Further, it was found that the SPCE gradually increased from 21 to 78% with decreasing stationary catholyte (SC) thickness from 250 to 65 μm. Fig. 3f showcases the SPCE values with respect to feed CO_2_ flowrate. Using the BPM electrode assembly configuration, the authors were able to achieve 200 mA cm^−2^ at 3.82 V full cell voltage, and at a corresponding C_2_H_4_ FE of 40% (Fig. 3e). For reference, an SPCE increase from 4 to 35% decreases the cathode separation energy from 85 to 15 GJ per ton of C_2_H_4_. Comparing this approach with conventional neutral pH eCO_2_RR, an approximately 30% reduction in overall energy is achieved.^104,105^ Advancements made by the Boettcher group in measurement platforms now allow for precise characterization of how the electric field, chemical catalysis, and interfacial water dynamics contribute to efficient and robust water dissociation and recombination in BPMs.^106,107^ Alongside these, the development of materials models that can predict the design of new WDCs and the creation of new non-precious-metal catalysts facilitates BPM operation near the thermodynamic limit at a technologically relevant current density of >1 A cm^−2^,^108,109^ thereby unlocking new applications in (bi)carbonate-mediated eRCC without the historically penalizing energy requirements. BPM advancement is still warranted to advance the understanding of their fundamental performance limits.^110^ It is essential to determine whether, with adequate ionic conductivity and efficient WDCs, performance can approach the thermodynamic limit, similar to the case with PEM water electrolysis. This involves constructing more complex ionomer multilayers to effectively control ion transport and local pH, utilizing the design of water dissociation layers. Additionally, tailoring the local anode and cathode environments with ionomers of different p*K*_a_ ranges, which provide varying internal pH values, will be crucial in optimizing performance.

An overarching goal of eRCC is the circumvention of the initial upstream CO_2_ purification and compression steps. Realistically, however, the literature at this stage is solely focused on short-term performance effects that act as direct evidence of beneficial electrolyzer configurations and catalyst designs. A typically concentrated bicarbonate electrolyte (3.0 M KHCO_3_) is used in the literature, whereby the regeneration step is typically overlooked since long-term operation has been neglected thus far. This is quite problematic for realistic TEA models that assume kW and MW capacity steady-state capacity production scenarios based on minute-scale FE experiments under ideal conditions. Furthermore, it is important to recall that point-source emissions – that would ideally be fed into concentrating the bifunctional capture liquid and electrolyte (*i.e.*, bicarbonate, amine, ionic liquid, *etc.*) – are effectively low concentration CO_2_ feed streams. It is unlikely that the conventional 3.0 M bicarbonate electrolyte can be achieved through direct transformation of flue gas into a hydroxide, at least without a large fraction of impurities. This is because given the same capture hydroxide solution, it would take almost 10 times longer to reach the same bicarbonate concentration starting from a 5 mol% CO_2_ feed compared to a 50 mol% CO_2_ feed. The longer ‘capture’ time is likely to increase the ratio of impurities as well. Therefore, to have a comparable setting with conventional eCO_2_RR, future work pertaining to (bi)carbonate-mediated eRCC should focus on prolonged experiments with realistic concentrations and compositions of (bi)carbonate. However, it is worth mentioning that the area of electrocoupling C–N to produce organonitrogen compounds through co-reduction of CO_2_ and NO_3_^−^/NO_2_^−^ as the N source is an active field.^111^ Therefore, although this is expected to be catalyst dependent and requires dedicated work to investigate, NO_*x*_ impurities in the bicarbonate electrolyte may result in a complete degradation of it. Preliminary work by Pimlott *et al.* reports SO_*x*_ and NO_*x*_ impurity effects in (bi)carbonate-mediated eRCC for a Ag catalyst targeting CO production.^112^ Interestingly, up to 2000 mg L^−1^ SO_*x*_ had no effect on the attained activity or selectivity. However, both NO_3_^−^ and NO_2_^−^ drop the FE towards CO from ∼60 to 5% upon introducing 2000 mg L^−1^. The authors showcased that 5 mM dodecyltrimethylammonium bromide (DTAB) is enough to sustain the original control FE towards CO due to the adsorption of DTAB on the catalytic surface as an amphiphilic surfactant to minimize competitive reduction reactions. It is important to note that this study, although timely, targeted only two impurities. The competing ORR was not investigated yet for (bi)carbonate-mediated eRCC. Further, although surfactant assisted durability in the presence of impurities is an option, this needs to be generalized across different catalyst types and target products. Temperature effects are recommended to be investigated at prolonged operating conditions as well, as they are expected to increase current densities due to more kinetically facile bicarbonate to CO_2_ dissociation but potentially at the cost of degrading cell components (*i.e.*, interposer).

## 4 Emerging solid-state adsorptive capture and conversion (ACC)

As was mechanistically highlighted for both amine and (bi)carbonate-mediated eRCC, it is the dissolved or *in situ* generated CO_2_ that is the effective reactant in both cases – at least under current density ranges typically achieved for both eRCC approaches. Furthermore, the capture and reduction steps are spatio-temporally exclusive. These limitations, coupled with lower current densities than those achieved by conventional eCO_2_RR, shine the spotlight on transport limitations being a pivotal factor in determining reaction rates. Emerging approaches using a hybrid solid GDE have been proposed and showcased, albeit to a much lesser extent compared to liquid-based eRCC, wherein pre-concentration of CO_2_ is achieved on the gas-side of the GDE prior to reaching the catalyst for direct CO_2_ reduction. These ‘pre-concentration’ layers can be generally divided into membrane-based and MOF-based layers which will be highlighted in this section. MOF-based GDEs are effectively grown on conventional carbon-based supports.

### 4.1 Mixed-matrix membrane-based GDEs for ACC

Conventional membrane separation methods leverage the differences in partial pressure and concentration of target gas (*i.e.*, CO_2_) across a membrane to facilitate the rapid transfer and accumulation of CO_2_ on the opposite side. The efficiency of this process is primarily governed by the selectivity and permeability of the membrane material towards CO_2_, but is also determined by durability under the operating environment, anti-plasticization, anti-aging properties, and low manufacturing costs. Compared to other post-combustion capture techniques, conventional membrane separation offers several advantages, including lower energy consumption, lower CAPEX, and a smaller environmental footprint.^113^ However, the commercial application of this technology is limited by the performance and stability of membrane materials. Research in the area of membrane-based gas separation has a thermodynamic degree of freedom that would typically be missing in eCO_2_RR, namely increasing the feed pressure of the mixed gas stream.^114^ Although contemporary catalyst designs and activities achieve hundreds of mA cm^−2^ current density under pure CO_2_ feed streams, the overwhelming majority of feed CO_2_ is wasted through bicarbonate formation or low SPCE. This still presents an opportunity for ambient pressure membrane-separation if each GDE has an independently integrated separation layer, membrane-based or otherwise. Gas separation membranes have been actively studied for over two decades, which resulted in several membrane groups, namely polymeric, inorganic, and mixed-matrix-membranes (MMM), each with their intrinsic properties featured towards certain applications. Of interest to electrochemical applications in general, and ACC in specific is the MMM type.

Briefly, MMMs entail the introduction of inorganic fillers as the dispersed phase in a polymeric matrix as the continuous phase, thereby captaining features of both inorganic and polymer type separation membranes. For instance, Amooghin *et al.* added 15 wt% of nanoporous NaY zeolite to Matrimid®5128 and reported a 16% increase in the gas permeability coefficient and 57% increase in CO_2_/CH_4_ selectivity.^115^ The gas separation performance of MMMs is influenced by several factors, including the compatibility between porous filler materials and polymers, the particle size of the porous fillers, and the interfacial morphology between fillers and polymers. Among these, interfacial morphology plays a crucial role in determining the separation efficiency of the membranes. To achieve optimal interfaces in MMMs, it is essential to ensure that the polymers and fillers are well-matched through both materials exhibiting preference towards the target gas as well as through minimization of the filler size so as to prevent blockages and mechanical defects within the membrane matrix. Briefly, two primary transport mechanisms of CO_2_ can occur in MMM: (1) the preferred facilitated transfer mechanism and (2) the dissolution–diffusion mechanism.^116^ In the facilitated transfer mechanism, the membrane contains a reactive carrier site that undergoes a selectively reversible reaction with CO_2_. In contrast, the dissolution–diffusion mechanism is strongly correlated with condensation and kinetic parameters of the gas considered and occurs through a three-step adsorption, active diffusion, and desorption process of unreacted CO_2_ through the membrane. Herein, the dissolution selectivity is usually modulated through functional polar groups that can enhance specific gas solubility. Analogously, gas type diffusion selectivity is a function of the pore structure of the fillers as well as the interfacial structure between the polymer and filler through which stronger interfacial interactions stiffen the polymer matrix on the filler surface. This effect can enhance both diffusion and solution selectivity.

To effectively activate the highest potential of MMMs, the compatibility and interaction between the host polymer matrix and the corresponding filler material needs to be rationally controlled so as to prevent the formation of non-selective defect sites. A host of material groups have been investigated for fillers in different polymers including, but not limited to, CNTs,^117^ carbon molecular sieves (CMS),^118^ MOFs,^119^ COFs,^120^ zeolites,^121^ and several 2D material-based families (*i.e.*, MXenes, graphene, graphene oxides, g-C_3_N_4_),^122–124^ and combinations thereof.^125^ A performance tradeoff can be thought to exist between the different fillers in terms of selectivity and permeability. For instance, high aspect ratio fillers such as CNT result in high diffusional pathways for gas permeability but potentially at the cost of lower interfacial contact with the polymer matrix. However, surface functionalization of fillers has been an effective strategy towards enhancing interfacial heterojunctions between the filler and polymer matrix. Ansaloni *et al.* demonstrated a CO_2_ permeability coefficient of 957 Barrer with CO_2_/N_2_ selectivity reaching 384% by utilizing NH_2_-functionalized CNTs within a polyvinyl alcohol–polysiloxane matrix.^126^ The work on MMM development towards gas separation is not new *per se*, and continues to witness research efforts. We refer the reader to two recent review articles by Niu *et al.* and Jia *et al.* on the topic.^116,127^

**Fig. 4:**
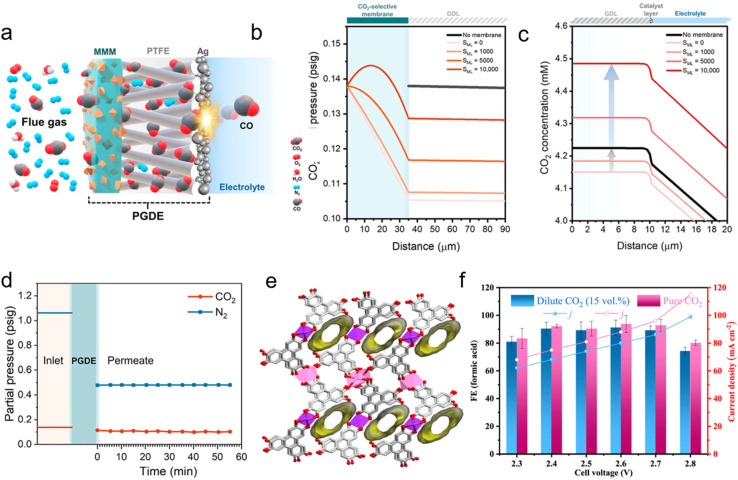
(a) A schematic representation of the permselective gas diffusion electrode (PGDE) showing the MOF-based mixed matrix membrane (MMM) on the gas feed side and sputtered Ag on the reaction side. (b) CO_2_ partial pressure through the CO_2_-selective membrane with different CO_2_ sink values that resemble the CO_2_ interaction with the membrane layer (*S*_ML_, mol (m^3^ s)^−1^) *versus* CO_2_ partial pressure without the membrane layer. (c) Concentration profiles for CO_2_ dissolved in the catholyte (1 M KHCO_3_) after permeating the membrane with different *S*_ML_ values *versus* CO_2_ permeated without the CO_2_-selective membrane. (d) Breakthrough results for the PGDE (7 mg cm^−2^ CALF-20 in the MMM layer). Feed conditions: 88.5% N_2_ and 11.5% CO_2_ (by gas chromatography), *P* = 1.2 psig, CO_2_ flow rate = 10 sccm, and N_2_ flow rate = 90 sccm. Reprinted with permission from ref. 128. Copyright 2022, American Chemical Society. (e) Coordination structure of the 3D π–π stacking structure of Bi-HHTP (HHTP = 2,3,6,7,10,11-hexahydroxytriphenylene) with 1D pores along the *b*-axis direction. (f) FEs of formic acid and current densities under different cell voltages in high-purity CO_2_ and dilute CO_2_ (15 vol%, CO_2_/N_2_ = 15 : 85, v/v) atmospheres, respectively. Reprinted with permission from ref. 130. Copyright 2024, American Chemical Society.

The integration between MMM and eCO_2_RR towards solid-state ACC is still an emerging approach with a mere handful of published work. Holistically overcoming many of the intrinsic challenges facing eRCC approaches, the advent of ACC is expected to be a popular approach towards direct flue gas reduction to value-added hydrocarbons and oxygenates alike. Furthermore, the coupling of MMMs and eCO_2_RR seems to be a natural use-case from the advancements of MMMs. Al-Attas *et al.* reported the first effective MMM type GDE as an ACC using a CALF-20 MOF filler directed towards impurity containing quasi flue gas (CO_2_/N_2_/O_2_ = 10–15 : 4 : balance, v/v/v, 100% relative humidity (RH)) electrolysis.^128^ Notably, a high loading (7 mg cm^−2^) of CALF-20, a known MOF for high CO_2_ sorption under humid conditions, was prepared with Nafion polymer, also favoring CO_2_ sorption, on the feed-gas side of a PTFE gas diffusion layer (GDL). Following this, 300 nm of Ag was sputtered on the reaction-side of the GDE. A representative schematic of the hybrid GDE configuration is presented in Fig. 4a. Since CO_2_ permeability and selectivity tradeoffs will naturally exist, COMSOL Multiphysics v6.0 was used to investigate the validity of the aforementioned hybrid GDE. The impact of membrane selectivity on CO_2_ separation has been analyzed using a mixture-averaged diffusion model for a CO_2_–N_2_ binary gas system. The findings indicate that a CO_2_-selective membrane significantly lowers the permeated partial pressure of N_2_, while only marginally reducing the CO_2_ permeate partial pressure, as shown in Fig. 4b. As the membrane becomes increasingly selective for CO_2_, the partial pressure of CO_2_ rises, leading to higher dissolved CO_2_ concentrations and enhancing reactant availability at the dual-phase reaction interface for CO_2_ reduction (Fig. 4c). Experimentally validating this, breakthrough experiments showed 1.1 × 10^6^ gas permeation units (GPUs) with a 2.1 selectivity of CO_2_/N_2_ with a feed stream of ∼10% CO_2_ in N_2_ balance. It can be seen that the partial pressure of N_2_ across the hybrid GDE significantly dropped from 55% compared to a slight decrease of ∼0.03 psig for CO_2_ partial pressure, resulting in a higher concentration of CO_2_ in the permeate. Electrochemical findings indicate that incorporating the CALF-20-based MMM layer increased the CO FE by approximately 64% compared to the control bare Ag/PTFE at −1.32 V (*vs.* RHE), from 95 to 58% for the control sample, when exposed to a diluted gas stream containing 10% CO_2_. Notwithstanding, the addition of 4% O_2_ to the dilute CO_2_ stream significantly affected the electrochemical performance of the bare Ag/PTFE electrode due to the prevalent parasitic oxygen reduction reaction. However, the introduction of the MMM layer inhibited the parasitic oxygen reduction reaction by increasing the current density from 7 to ∼30 mA cm^−2^.

### 4.2 MOF-containing hybrid GDEs in ACC

Although not explicitly considered MMM-based GDEs, there has been some work dedicated to flue gas CO_2_ electrolysis as eluded to earlier which is worth highlighting. In those reports, a high CO_2_ affinity MOF or COF is used both as the selective sorbent and active region for reduction.^129^ This adds the benefit of lowering the number of layers in the GDE which can correspondingly lower Ohmic losses and transport limitations. However, this also adds a layer to the catalyst/sorbent design considerations wherein durability and selectivity under adsorption and reduction need to be considered and studied, without affecting catalytic activity. That being said, Liu *et al.* recently showcased high FE towards CO under 120 mA cm^−2^*via* the reduction of synthetic model flue gas composition of CO_2_/N_2_ (15 : 85, v/v) under high humidity.^129^ This was accomplished using a Ag_12_ nanocluster-based MOF, namely [Ag_12_-(S^*t*^Bu)_8_(CF_3_COO)_4_(3-amino-4,4′-bipyridine)_4_] which further showed maintenance of the aforementioned FE and current density for 300 hours of operation corresponding to EE and SPCE of 56 and 60%, respectively. Other sorbent/catalysts were tested using the same MOF, but under different linkers. The 3-amino-4,4′-bipyridine was replaced in one instance with a 3-amino-4,4′-bipyridine and in another with just the 4,4′-bipyridine linkers. Interestingly, the effect of functionalization on the MOF was notable, whereby the samples tested without amino functional groups showed expectedly low activity towards eCO_2_RR under flue-gas composition. This phenomenon confirms the so-called ‘CO_2_ relays’ effect the amino group has towards directing CO_2_ towards Ag active sites for reduction. The high tunability of MOFs resulted in the formulation of over 20 000 structures thus far. Therefore, modulating linkers and active sites in MOFs is not considered very challenging. Emerging efforts in this direction of direct flue-gas reduction on functionalized MOFs are on the rise. Zhao *et al.* recently targeted formic acid through the direct reduction of simulated flue gas with realistic impurities (CO_2_/N_2_/O_2_ = 15 : 80 : 5, v/v/v) and through high humidity (40% RH) through using a microporous Bi-based MOF (Bi-HHTP, HHTP = 2,3,6,7,10,11-hexahydroxytriphenylene).^130^ A high conductivity (0.53 S m^−1^) exists for the Bi-HHTP, due to the 3D π–π stacking shown in Fig. 4e, allowing for reasonable charge transfer resistance during electrolysis. This material not only exhibited promise towards eCO_2_RR under low feed concentrations, but in the presence of O_2_ as a primary damaging impurity that tends to sway the selectivity towards the ORR as a competing reaction. Bi-HHTP sustained 30 hours of operation at 2.7 V full cell voltage in the above-mentioned synthetic flue gas feed and achieved a stable current density of ∼80 mA cm^−2^ with around 90% FE towards formic acid (Fig. 4f). This resulted in a constant production of 200 mM aqueous formic acid solution with a relative purity of 100% as determined by ^1^H-NMR. Isotopic mapping confirmed that the resultant formic acid was coming from the isotopically labelled ^13^C in feed flue gas. However, it can be seen that when comparing performance metrics of MMM-based GDEs with non-MMM GDEs towards the direct reduction of flue gas feed streams, the former attains higher activities, durability, selectivity, and degrees of freedom towards modification and improvement. Moreover, Chen *et al.* recently investigated the effect of poly(4-vinylpyridine) (P4VP)-modified GDEs on dilute (10% CO_2_) feed streams, using several known CO_2_-to-CO catalysts including cobalt(ii) phthalocyanine (CoPc), Ag nanoparticles, and Ni–N–C type SACs.^131^ The GDL of choice was interestingly an in-house prepared 40 wt% PTFE-coated carbon paper prepared through sequential dip-coating and air calcination. P4VP and the respective electrocatalysts in different ratios were dispersed in an ink and spray coated on the PTFE-coated GDL. The P4VP-modified CoPc sample exhibited 90% FE towards CO under 10% feed CO_2_ conditions at a corresponding partial current density of 252 mA cm^−2^, which is a 2.24-fold increase compared to a control CoPc sample. The same material attained ∼35 hours of stability at 100 mA cm^−2^ using the dilute feed stream. Interestingly, this behavior was not limited to CoPc as the active molecular catalyst center, but also followed for the M–N–C and the nanoparticle materials as well. Integration of molecular catalysts with modifiers containing a high ratio of pyridine character resulted in a microenvironment that induced CO_2_ capture from dilute streams, followed by its reduction in neighboring electroactive sites.

The remaining handful of other work pertaining to MMM or MMM-like GDEs operates without flue gas composition as feed, instead using the conventional pure CO_2_ feed streams. For instance, a collaborative effort between Sargent and co-workers used a MOF-modified PTFE-based GDE whereby either an HKUST-1 or SIFSIX-3-Cu MOF was sandwiched between a PTFE substrate and a catalytic Cu overlayer.^132^ The goal of this work was to demonstrate the effect MOFs can have on concentrating the local CO_2_. To that end, the control PTFE/Cu GDE attained C_2_H_4_ FE of 43% and 30% at corresponding total current densities of 200 and 400 mA cm^−2^, respectively, showcasing local CO_2_ availability limitations at higher current densities. Adding a carbon adlayer and a cationic Nafion ionomer on the Cu layer increased the C_2_H_4_ FE to 44% at 400 mA cm^−2^, with performance enhancement being attributed to easier transport of the CO_2_ to active sites and more facile *CO formation at lower applied potentials. Upon addition of 0.6 mg cm^−2^ of calcined HKUST-1 between the PTFE GDL and the Cu/C top layer (C/Cu/HKUST-1/PTFE), the C_2_H_4_ FE increased from 43 to 51% at current densities from 400 to 525 mA cm^−2^. Furthermore, the hybrid GDE sustained C_2_H_4_ FE above 48% up to 1 A cm^−2^, nearly a two-fold enhancement compared to the MOF-free GDE in terms of C_2_H_4_ production rate which is attributed to the increased local availability of feed CO_2_ from the MOF. Although these results are quite promising towards C_2+_ production using a MOF-modified GDE, it remains to be seen whether such configurations will be effective towards the direct reduction of dilute CO_2_ in flue gas composition.

In a similar vein, it is worth noting that conventional cathodic electrocatalysts have been amine-functionalized towards enhanced retention of CO_2_ under constraining concentrations.^133^ Although we consider this to be a form of enhanced eCO_2_RR instead of integrated adsorptive capture and conversion (ACC) on a single solid-state GDE, their utilization in hybrid GDEs is warranted. Amino groups as catalytic surface modulators enhance not only surface CO_2_ adsorption,^134^ but also interfacial charge transfer and preferential pathways towards target products.^135,136^ Briefly, amine-functionalized cathodes tend to focus on enhancing C–C dimerization due to enhanced adsorption of CO and CHO reduction intermediates, which is known to increase the rate of C–C coupling to C_2+_ products.^137^ Notwithstanding, C_1_ target products such as CH_4_ have been shown to be selectively produced through amine-functionalized catalysts through amine-groups facilitating the hydronation step as well as stabilizing the CO intermediate.^138^ Recalling that most C_2+_ products follow the *CO intermediate pathway, it makes sense to rationally investigate combinations of a highly permeable and selective CO_2_ material (*i.e.*, MOF, MMM, *etc.*) used either behind (gas side) or directly atop the GDL substrate (sandwiched between the GDL and catalyst). Further, tapping into recent discoveries made in CO_2_ tandem electrocatalysis can be beneficial towards C_2+_ products under dilute CO_2_ feeds. In using a tandem catalysis architecture alongside the above-mentioned CO_2_ selective permeation layer, the hybrid GDE would be composed of four layers – with the gas-side facing two layers not being electroactive or contributing to the Ohmic overpotential. Therein, the first catalyst (above the two electrochemically inert layers) may contain amine-functionalized surfaces as a secondary layer of CO_2_ concentration and conversion to CO, followed by dimerization on a secondary Cu-based surface. Considering the plethora of C_1_ and C_2+_ catalysts developed to date, their compatibility in gradient layers or sequential adlayers in a GDE is recommended to be investigated towards flue gas ACC. Further, the utilization of appropriate ionomers in each of the above-mentioned steps needs fundamental electrochemical insights. To that end, Henckel *et al.* recently investigated the ionomer chemistry influence as well as the relative humidity (RH) of feed gas on cell performance during dilute CO_2_ electrolysis.^139^ Ionic resistance and electrocatalyst capacitance values for the cathode catalyst layer were evaluated as functions of RH and then correlated with CO FE and electrocatalyst utilization. It was determined that maintaining a CO_2_ concentration above 20% with at least 50% RH in the cathode resulted in CO/H_2_ selectivity exceeding 95%, regardless of the ionomer chemistry used. However, at a lower CO_2_ concentration of 10%, achieving over 95% CO/H_2_ selectivity required an RH of 95% and an electrode morphology that ensured high catalyst utilization. The role of ionomer chemistry in ensuring high electrocatalyst utilization was investigated and it was found that anionic imidazolium-based ionomer XA-9 – similar in structure to Sustanion – supported the highest CO selectivity under the dilute CO_2_ conditions used with a high-loading Ag (3 mg cm^−2^) catalyst. Although XA-9 exhibited the lowest ion-exchange capacity (0.94 mM g^−1^) compared to other anionic ionomers investigated in the study, it is believed that ionic polymer conformation plays a role in ion transport through the catalyst layer. It is worth noting that ionomer conformation has been reported to be affected by the method of ink preparation and deposition. The optimizations made in the study by Henckel *et al.* are non-universal and to a large degree dependent on the catalyst–ionomer–electrolyte-product combination, warranting further fundamental research on other catalyst–ionomer–electrolyte-product systems.^140^

## 5 Technoeconomic appraisal of technology-product couples

Several reports of integrated eRCC seem to omit critical design and operating aspects of both upstream feed CO_2_ as well as process-dependent factors that greatly influence the resultant quality of products – a crucial factor should these products and byproducts meet market standards. For instance, many studies to date tend to use a CO_2_ purchasing value of about 40 USD per ton whilst claiming that said CO_2_ can be sourced from DAC technologies – a far cry from the accepted range of DAC CO_2_ capture costs being greater than 200 USD per ton.^141,142^ The apparent literature benchmarked 40 USD per ton value of CO_2_ is intended to reflect negative CO_2_ pricing due to the 2008 introduction of the 45Q tax credit by the U.S. Internal Revenue Service (IRS), enactment in 2018 by the FUTURE Act, and the 2022 Inflation Reduction Act which contained significant enhancements to the 45Q tax credit. However, this value does not accurately represent the actual cost that would be paid through CO_2_ off-take agreements and may not be comparable to or relevant to the rest of the world. Moreover, many reports show a notable disparity in sensitivity analyses performed between C_1_ and C_2+_ products when considering variations in the CO_2_ cost.^142^ High TRL upstream CO_2_ separation and concentration technologies from point-sources (*i.e.*, amine scrubbing) offer the needed capacity for centralized CO_2_ electroreduction facilities, as well as offering a fair technoeconomic comparison when used for considering CO_2_ costs between different eCO_2_RR technologies. Briefly, process costs between 50 and 150 USD per ton of CO_2_ are expected for amine scrubbing with over half the cost being dependent on compression and regeneration of amine.^38^ Moreover, it can be often seen that simplified process diagrams are used during TEAs. For example, more than one gas-separation unit (*i.e.* pressure-swing absorbers (PSAs)) is needed if more than two gases exit the cathode side of the reactor. Should the product and byproduct be considered for the commercial market, their purity should be ensured. Similarly, if the anodic oxygen product is to be sold or if the crossover carbonate is to be recycled upon its oxidation to CO_2_, then an anodic gas-separation unit should also be considered. The same analogy can be made for cathodic liquid products.

Other aspects that are typically understudied entail separation costs of product(s) from liquid-based eRCC systems. This is expected to be exacerbated when target products are liquids, which would likely add significant costs to their minimum selling price (MSP) and take away from the holistic advantage of reactive capture media in terms of direct re-circulation or recycle for recapture of CO_2_. Furthermore, as is typically a major limitation of TEAs, intrinsic best-case optimism is considered for the TEA's base-case scenario. For instance, stability durations of emerging eRCC technologies, irrespective of target reaction-product couples, are considered irrelevant compared to the conventional sequential CO_2_ capture, compression, and reduction scheme. Yet, for the sake of attempting to grasp the TEA, 20 year plant models will be constructed and assumed to be operable with cell-component replacements considered on the timescale of years – taking from the more mature green water electrolysis field.^29^ Although this is an understandable practice to grasp, it shall be highlighted that the status-quo is far from the TEA models' base assumptions and that technical challenges be resolved by the time of deployment.

Understanding the fundamental relationships between key cell parameters, including cell potential, Faradaic efficiency, stability, single-pass conversion efficiency, and downstream separation costs, is critical for optimizing electrochemical processes. However, these parameters are not only interdependent but also highly dependent on the target product and the specific technology employed, making it essential to tailor strategies based on both the desired outcome and the system in use. In any electrochemical reaction, the energy cost is dictated by two primary factors: the number of electrons transferred per mole of product and the cell potential required to drive the reaction. Both of these factors play crucial roles in determining the overall energy efficiency and economic viability of electrochemical processes in general, and eCO_2_RR specifically. For example, in eCO_2_RR to C_2+_ products using a copper-based catalyst, the cell potential and current density are directly proportional, as higher current densities generally require higher cell potentials to maintain the reaction rate. However, this increase in current density and cell potential is not necessarily proportional to an increase in FE towards a specific C_2_ product like C_2_H_4_. In fact, while higher current densities can increase the rate of CO_2_ conversion, they can also lead to side reactions which compete with the desired product formation. As a result, the Faradaic efficiency for C_2+_ products can drop, even as cell potential and current density increase, highlighting the complexity of scaling up these reactions for efficient and selective product formation. Similarly, catalysts targeting C_1_ products, such as silver (Ag) or nickel (Ni)-based single-atom catalysts designed to electrochemically reduce CO_2_ to CO, face the same aforementioned challenge. A decrease in FE for C_1_ products often leads to increased downstream separation costs. This is because lower FE means that more by-products are formed, requiring additional purification steps to isolate the desired CO product. Moreover, as the efficiency drops, parasitic reactions such as the undesired HER, can become more prevalent, leading to increased wetting and flooding of the GDE over time, which causes the performance to deteriorate and adds to the operational maintenance costs of the electrolyzer. This interplay between FE, side reactions, and purification costs underscores the importance of optimizing both the catalyst and operating conditions to minimize these inefficiencies.

On the other hand, SPCE plays a significant role in determining the overall separation costs downstream. High conversion efficiency reduces the amount of unreacted CO_2_ that needs to be recycled, thus lowering the cost of separation. However, a recent study by Moore *et al.* highlighted that the energy required for the electrolyzer (cell potential) often dominates the separation costs.^143^ In their work, focusing on C_2_H_4_ production as a target product, they found that the optimal single-pass conversion efficiency for C_2_H_4_ is relatively low, around 5–10%. This approach helps maintain near-optimal electrolyzer performance and lowers overall production costs, suggesting that the energy demands of the electrolyzer should be carefully balanced with the target product yield to achieve the most cost-effective process. Moreover, in scenarios where the levelized cost of electricity (LCOE) is high or when cell durability is in question at high current density operation, it may make economic sense to lower the cell voltage and therefore current density. This would however require larger or more electrolyzer stacks to meet the production target, assuming FE for the target product is the same at the initial high and the modified lower cell voltage.

Keeping this in mind, we have formulated a net present value (NPV) model to calculate the minimum selling price (MSP) for several products, including CO and HCOOH, and under the considerations of different technologies. Cell-level details and specifications used for the technology-product couple considered are presented in Tables S1–S3.† As can be seen from the process flow diagrams (PFDs) (Fig. 1) we employed all process equipment and conditions to construct the TEA models. An exhaustive breakdown of the TEA models is presented in the ESI.† Briefly, and for a fair comparison between the examined technology-product couples, we took hydrogen (H_2_) as the only byproduct at a cathodic FE equaling 100 minus the FE of the target product. To that end, for each technology-product couple, two scenarios were assumed in attaining the final cumulative present value (CPV) – namely considering or not considering the revenue stream from selling byproduct H_2_. Irrespective of both scenarios, however, the corresponding capital (CAPEX) and operating expenditure (OPEX) of the H_2_ separation and purification were considered. This is partly because unreacted CO_2_ from the catholyte was recycled, and therefore needed to be separated out from both the byproduct H_2_, and the target product if it was gaseous (*i.e.*, CO, C_2_H_4_). For gas separation, pressure-swing absorbers (PSAs) were employed and for separation of liquid products a distillation column was empirically sized and modeled. All liquid products considered were modeled to be recovered from 30 wt% solutions. It is worth noting that the data used to model cell performance for all technology-product couples is based on top performing electrocatalysts in recent literature that have been examined with a corresponding technology. Four technologies, henceforth referred to as Systems, were considered in our TEA investigation. These include the conventional sequential CO_2_ amine-based capture, stripping, compression, and electrochemical reduction (System 1), amine-mediated eRCC (System 2), (bi)carbonate-mediated eRCC (System 3), and direct flue gas reduction through ACC through MMMs (System 4). In all base-case models considered, a literature convention of 100 tons per day production capacity of the target product was modeled. Further, base economic assumptions employed in the developed TEA model include a 20 year plant lifetime with 350 operating days per year, a 38.9% income tax, 10% discount rate, and a modified accelerated cost recovery system (MACRS) of 10%.

### 5.1 Carbon monoxide and formate/formic acid as target products

Early TEA studies that targeted the field of eCO_2_RR showed remarkable promise towards the profitability of conventional sequential CO_2_ capture, concentration, compression, and electrochemical reduction. However, the advent of different integrated capture-reduction routes (*i.e.*, amine and (bi)carbonate eRCC) led to dedicated TEA investigations aimed at comparing the holistic energetic benefits between eRCC and conventional sequential eCO_2_RR. Commenting on the early work by Jouny *et al.* in 2018, ∼100 MW (100 tons per day production rate) plants operating at 200 mA cm^−2^ current density having base-case scenario cell voltages of 2.3 V, product FEs of 90%, and SPCE of 50% – performance parameters that are still considered difficult to achieve for some products – showcased that only CO and HCOOH were considered profitable under the net-present-value (NPV) model.^142^ Under an optimistic scenario, wherein 300 mA cm^−2^ could be sustained at a cell voltage of 2.0 V, ∼60% reduction in the LCOE and purchase price of CO_2_, respectively, compared to the base case, it is found that high-order oxygenates like *n*-propanol are most profitable. It can be deduced that for C_2+_ oxygenates and hydrocarbons to be economically meaningful for targeted production, the natural lowering of LCOE and required voltage, as well as ensured stability at high selectivity values need to be maintained. Although the conclusion of such work seemed promising, the technicality needed to achieve 90% FE towards C_2+_ products at low cell-voltages (<2.5 V) at commercially relevant current densities (200–400 mA cm^−2^) is presently considered challenging in lab-scale demonstrations, which becomes exacerbated due to simplifications on the side of product gas/liquid separation technologies made during TEAs.

Converting current CO_2_RR product streams into marketable products necessitates extensive downstream separation processes. Energy analyses indicate that low product concentrations can result in separation energy surpassing the electricity input needed by the electrolyzer, both for gaseous and liquid products. On that note, a dedicated study by Sinton and Sargent groups found that downstream CO_2_ separation is the most energy intensive step in a CO_2_ to C_2_H_4_ electrolyzer.^80^ It was found that to reduce the separation energy to approximately 22 GJ per ton of C_2_H_4_, FE towards C_2_H_4_ needs to exceed 57% in a gas-only production cell, SPCE needs to exceed 80%, and a configuration that prevents carbonate crossover is to be used. For reference on the importance of carbonate formation, it was estimated that 534 out of 576 GJ per ton of C_2_H_4_ is attributable to CO_2_ separation from the anode upon carbonate crossover – roughly 1.6 times the energy of the actual electrolysis step. Moreover, the presence of minor components within streams will further escalate the costs and complexity of separation processes.^144^ Efforts by Greenblatt *et al.* further demonstrate the degree of complexity needed for realistic separation of target products from electrolyzer tail-gas streams.^145^ Therefore, finding appropriate methods to fine tune selectivity and effluent, or tail-gas, concentrations of the target product is fundamentally critical to the feasibility of the overall process, independent of the eCO_2_RR approach employed.

The primary reasons why HCOOH and CO are generally considered profitable with existing cell performance are high FE (>90%), high current densities (>200 mA cm^−2^), and low cell potentials in MEAs (<2.0 V). Briefly, the majority of C_1_ and C_2+_ products undergo the *CO pathway during CO_2_ reduction, irrespective of whether this is performed under conventional sequential capture-eCO_2_RR or eRCC routes.^146^ Further, several transition metal (TM)-based electrocatalysts have been developed and studied thoroughly through *in situ* and computational methods in targeting the two-electron transfer CO production under industrially relevant conditions.^147,148^ Coupling this with the mechanistic challenge and catalyst selectivity towards forming other C_1_ or C_2+_ gas products, the separation of CO is relatively facile from the cathode side – with the main challenge being unreacted CO_2_ during low SPCE – an issue which is largely addressed in eRCC approaches. Limiting the feed CO_2_ flowrate into the cathode side can limit unreacted CO_2_, however at a trade-off of increasing the risk of undesired HER.^149^ Thermodynamically speaking, however, increasing the feed pressure of low flow-rate CO_2_ can be envisioned to lower the competing HER as well as unreacted CO_2_, making downstream processing of CO more tangible and adding the benefit of pre-pressurizing the target CO product.

Similarly, for the liquid C_1_ target product with existing economic feasibility, namely HCOOH or primarily formate (HCOO^−^), the use of certain TM-based catalysts (*i.e.*, Sn, In, Bi, Pb) practically offers a mechanistic limitation towards other CO_2_RR products, thereby offering >90% FE towards HCOO^−^.^150^ Investigations towards the use of AEMs, CEMs, and BPMs in conventional MEA offer a TEA opportunity towards qualitatively understanding of how to best increase the concentration of target liquid products. For instance, in the case of typical AEM-based MEA configurations 15 wt% (3.5 M) HCOO^−^ has been reported.^151,152^ Notwithstanding, achieving higher rates of concentration in such cell configurations is practically challenging due to the natural flow of anionic HCOO^−^ towards the anolyte side through the AEM, where re-oxidation to CO_2_ is possible, while diffusion and electro-osmotic drag occurs for neutral charge products such as alcohols.^153^ Although CEM configurations prevent both carbonate and HCOO^−^ crossover to the anolyte, they add a layer of challenge pertaining to generating a local acidic micro-environment at the cathode which favors undesired cathodic HER. An alternative approach, which we have previously discussed in detail, is utilizing BPM configurations. This approach, if properly employed and optimized, offers the protonation of HCOO^−^ to its more market-valuable carboxylic acid counterpart (HCCOH) in the catholyte, and simultaneously prevents carbonate crossover. Using novel WDC BPMs is expected to significantly lower the conventional high overpotential known for water dissociation in BPMs, whilst offering the same intrinsic advantages offered in a BPM cell configuration. For reference, liquid products (*i.e.*, C_2_H_5_OH) are typically diluted to <0.1 wt% in the catholyte and the energy requirements for separating 1.5 wt% of C_2_H_5_OH exceeds the lower heating value of the alcohol.^145^ On the note of concentrating liquid products prior to downstream separation, namely to their more valuable carboxylic acid analogues, solid-state electrolytes (SSEs) have been utilized to generate high concentrations of target liquid products (∼50 wt% (12.0 M) HCOOH).^154^ Briefly, in an SSE cell an ion-conducting functionalized polymer SSE (*i.e.*, sulfonic acid functionalized styrene–divinylbenzene copolymer) is sandwiched between an AEM catholyte and a CEM anolyte, whereby protons from anodic water oxidation travel through the CEM and protonate HCOO^−^.^155^ SSEs are known to add high Ohmic losses, thereby increasing the needed cell voltage to sustain a certain current density, and their long-term stability under eCO_2_RR remains to be tested.

Li *et al.* adopted commercial MEA (30 wt%) as a CO_2_ capture solvent and performed a comparative energetics study between the conventional sequential approach and the amine-mediated eRCC towards CO production.^156^ Briefly, it was calculated that 643, 254, 51, and 179 kJ mol^−1^ of CO_2_ would be needed towards electrolysis, bicarbonate regeneration, product purification, and upstream amine regeneration in the scrubbers, respectively, assuming 50% CO_2_ utilization. As disclosed earlier, the high transport limitations in amine-mediated eRCC dictates a higher applied voltage to reach a given current density. To that end, it was found that under the baseline scenario for amine-eRCC, electric energy alone amounts to 1103 kJ mol^−1^ of CO_2_, compared to 733 kJ mol^−1^ of CO_2_ needed for electric energy in the conventional sequential approach. An optimistic scenario envisioning 90% instead of 70% FE towards CO, and 3 V instead of 4 V for the integrated approach, results in a 44% energy saving. To that end, energy costs alone – without accounting for capital expenditure (CAPEX) – yielded 278 (base-case) *versus* 203 USD per ton of CO for the integrated *versus* sequential route, respectively, and a further drop from 278 to 162 USD per ton of CO under the optimistic model. Similarly, Lee *et al.* performed a comparative TEA on flow-cell and MEA type cells using sequential eCO_2_RR, as well as on direct carbonate and direct amine eRCC towards CO production.^53^ Their findings highlight that although direct eRCC technologies result in no carbonate formation, crossover, and thereby no impurity or unreacted CO_2_ in the outlets and no CO_2_-associated regeneration costs from upstream regeneration of capture agents, the total energy requirements for the conventional methods are quite close to that of eRCC for CO production. For instance, total energy requirements of 834, 814, 2597, and 668 kJ mol^−1^ of product were needed for flow-cell, MEA, direct carbonate eRCC, and direct amine eRCC, respectively. The primary cost in both eRCC approaches is attributed to the electrolysis energy needed – again due to the limiting auxiliary components (BPMs in carbonate eRCC) and feed CO_2_ (or carbamate) transport limitations of current liquid-based eRCC technology.

**Fig. 5:**
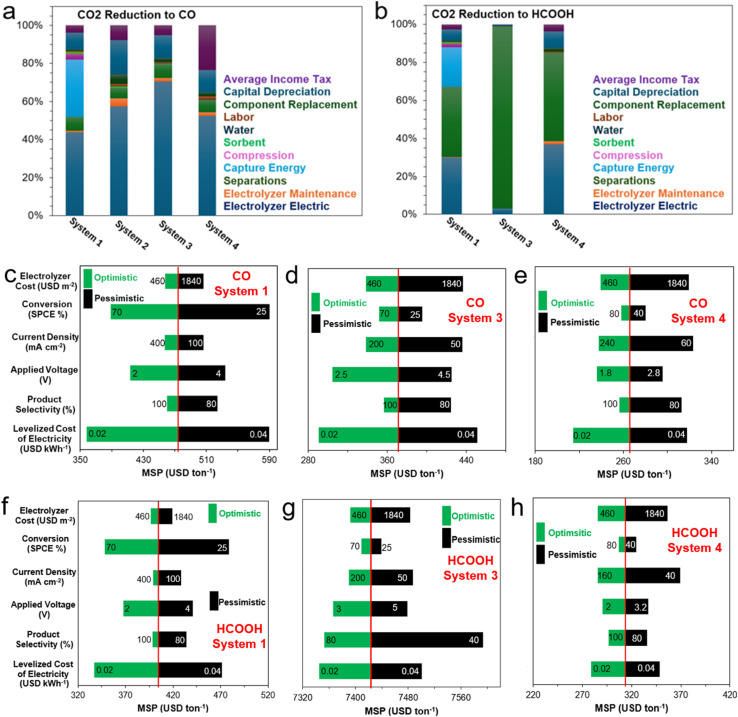
(a) System-dependent OPEX breakdown for 100 tons per day CO and (b) HCOOH production. (c and f) Sensitivity analysis of System 1 (conventional sequential CO_2_ capture-eCO_2_RR), (d and g) System 3 ((bi)carbonate-mediated eRCC), and (e and h) System 4 (direct ACC) for (c–e) CO and (f–h) HCOOH.

As can be seen in Fig. 5a for CO production, irrespective of the systems investigated the greatest OPEX component is indeed the electrolyzers' electricity requirement. Approximately 30% of the cost is allocated to upstream energy requirements for CO_2_ capture in System 1, whereas other systems do not incur this cost as upstream CO_2_ capture is not required. The separation component, namely PSAs since CO is a gas, is consistently around 6–7% of the OEPX for all systems. At an NPV of $0 for the case of CO as a target product, the corresponding MSPs for Systems 1 through 4 are 473.7, 452.9, 370.8, and 266 USD per ton of CO in the scenario where the revenue stream of purified byproduct H_2_ (and anodic O_2_ (g)) is not considered. Since the commercial price for purified CO is approximately between 600 and 720 USD per ton, the status quo of all four technology options is economically viable. Although the direct ACC model (System 4) showcases a notable 44% production cost reduction compared to the conventional route (System 1), the stability and impurity agnostic aspects of the MMM GDEs used in System 4 need to be confirmed. A similar OPEX breakdown was undertaken for HCOOH as an economically feasible target product (Fig. 5b). It should be noted that since no reports of HCOOH have been suggested under amine-mediated eRCC (System 2), it has been omitted from consideration. Again, a large component of the OPEX is attributed to the electrolyzers' energy requirements. However, for the case of HCOOH as a liquid product a notable change in cost is attributed to separations – namely distillation. As shown in the relevant PFDs in the ESI† that both distillation columns and PSAs are employed for liquid and gas separation, respectively, the OPEX component of distillation is substantially higher than that of the PSA. Briefly, about 7.5 and 2.2% of the separation OPEX is from PSAs in Systems 1 and 4, respectively. This is a far cry from the CAPEX comparison, whereby 64.5 and 37.5% of the CAPEX separation cut is from PSAs for the same Systems 1 and 4, respectively. The use-case for PSAs in liquid target products (*i.e.*, HCOOH) includes unreacted CO_2_ purification and recycle from catholyte effluent streams, H_2_ (g) byproduct separation, and anodic O_2_ separation from CO_2_, wherein CO_2_ is a result of oxidation of the crossed-over carbonate in System 1. As tabulated in the ESI,† setting the NPV to $0, MSPs of 402.2, 7422.5, and 313.7 USD per ton of HCOOH corresponding to Systems 1, 3, and 4, respectively, are attained when revenue streams from H_2_ and O_2_ byproducts are not considered. The order of magnitude larger MSP corresponding to System 3 is predominantly due to the need to separate the liquid HCOOH from the liquid (bi)carbonate capture agent. This results in about 92.4% of the corresponding blue-colored 96% Separations OPEX cut shown in Fig. 5b. As will be seen in later analyses of System 3 with target liquid products, these impractical separation requirements of the liquid product-capture agent couple are a running theme and critical bottleneck of both amine- and (bi)carbonate-mediation-based eRCC systems. Tabulated OPEX breakdowns for all the considered technology-product couples are presented in Tables S4–S8.†

Sensitivity analyses in Fig. 5c and f tend to draw the picture that for both CO and HCOOH under the conventional System 1 design, levelized cost of electricity (LCOE), applied cell voltage, and conversion are the three primary factors that affect the attained MSP which is in good agreement with the literature. For liquid-based eRCC, sensitivity analyses in Fig. 5d, e, g and h show that although the effect of LCOE and applied voltage still on average dominate the resultant MSP, current density starts playing an important role as well. This is expectedly due to the relatively low base-case current density presented in contemporary literature (about 100 mA cm^−2^) for both (bi)carbonate eRCC and ACC to attain acceptable product selectivity. Generally, it can be seen that the area normalized capital cost of the electrolyzer cells and the single-pass conversion efficiencies only play significant roles on the MSP if the aforementioned three parameters are high enough. As was alluded to earlier however, effectively changing one cell operating parameter will likely tend to change others. For example, the discovery of more conductive catalysts that require less voltage to achieve the same current density may not necessarily exhibit the same FE, thereby dictating more electrolyzer stacks to meet production requirements. Similarly, a temporary increase in the LCOE may shift the economics towards lower operating voltages under the same catalyst, which would translate to lower current densities and a potentially higher or lower FE depending on the reaction. In contrast, a lower LCOE may compel operation at higher voltage and current density at the risk of a lowered catalyst durability that requires replacement more often. Moreover, for eRCC (Systems 2 and 3) and ACC (System 4) technologies, which are less mature compared to the conventional SCCC (System 1), the achievable current density is generally much lower. For example, while the electrolyzer cost has a minimal impact on the cost sensitivity of System 1 for CO production (Fig. 5a), it becomes a dominant factor in the amine-mediated eRCC sensitivity analysis (Fig. S1†). This disparity arises because the base-case partial current density for amine-mediated eRCC is 5.3 times lower than that assumed for the conservative System 1 calculation in Fig. 5a. Consequently, a linear increase in electrolyzer area—5.3 times larger—is required for System 2 to achieve the same target production rate of 100 tons per day, making electrolyzer costs a more significant contributor to the MSP under the constraints of amine-mediated eRCC. Ultimately, these factors must be evaluated with careful consideration of their interdependent trade-offs for a specific catalyst-reaction system, rather than being generalized across different reactions.

### 5.2 Alcohols and ethylene as target products

**Fig. 6:**
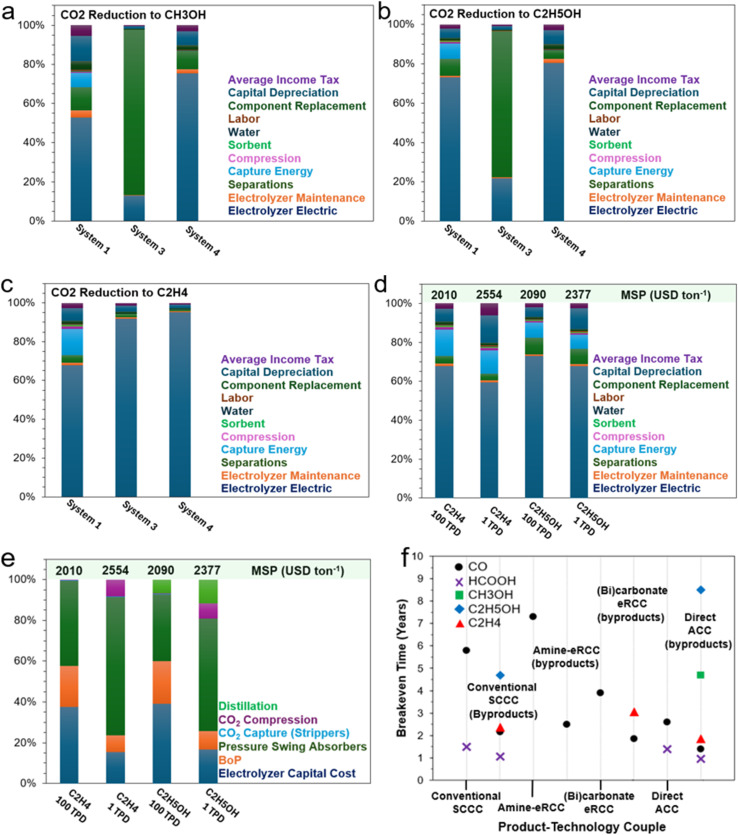
(a) System-dependent OPEX breakdown for 100 ton per day CH_3_OH, (b) C_2_H_5_OH, and (c) C_2_H_4_ production. (d) Production-scale dependent OPEX and (e) CAPEX breakdown of C_2_H_5_OH and C_2_H_4_ production. (f) Breakeven time requirement for techno-economically feasible technology-product couples.

Early work by Jouny *et al.* employed an NPV model with optimistic-case LCOE of 0.02 USD per kW h and 1.7 V cell voltage requirements at 500 mA cm^−2^ current density and 100% target product selectivity, 70% SPCE, and a literature standard of 920 USD per m^2^ for area normalized electrolyzer costs to yield an NPV of 25 million USD for ethylene under the governance of the conventional sequential CO_2_ capture, compression, and reduction approach (System 1).^142^ This is based on a 100 tons per day production capacity of ethylene. Therein, an optimistic fully subsidized CO_2_ input was assumed. Similar and more recent TEA efforts by Jing *et al.* using a more realistic fixed optimized purchasing cost of 30 USD per ton of CO_2_, 2.5 V cell voltage to sustain 500 mA cm^−2^ current density with 60% target product selectivity, 25% SPCE, and an LCOE of 0.03 USD per kW h yielded levelized costs of 1335, 1795, and 2839 USD per ton for methanol (CH_3_OH), ethanol (C_2_H_5_OH), and ethylene (C_2_H_4_), respectively.^157^ As can be seen from the ESI,† the base-case MSP values for CH_3_OH, C_2_H_5_OH, and C_2_H_4_ are 1472, 2089, and 2009 USD per ton, respectively, for System 1 in the scenario where revenue streams from byproduct H_2_ and O_2_ are not considered in the NPV model, offering comparable results with recent literature. Notwithstanding, since we considered eRCC and direct ACC systems in this work, the corresponding OPEX breakdown for CH_3_OH (Fig. 6a), C_2_H_5_OH (Fig. 6b), and C_2_H_4_ (Fig. 6c) is presented. It can be clearly seen that much like C_1_ products in Fig. 5, electrolyzer electricity requirements take the overwhelming share of the operating costs, followed by separation energy requirements – and upstream capture energy requirements for System 1. Further, for target liquid products under System 3 ((bi)carbonate eRCC), namely CH_3_OH and C_2_H_5_OH, separation costs increase disproportionately due to the need to separate said liquid products from the liquid-based capture solution. Further, due to the 12-electron step CO_2_ reduction to C_2_H_5_OH and C_2_H_4_, the electricity requirements to the electrolyzer are substantially larger than those of the C_1_ hydrocarbon or oxygenate counterparts. It is worth noting that base-case analyses performed herein were for target product production capacities of 100 tons per day (TPD), as is typically examined in the literature assuming a centralized facility. However, Fig. 6d and e compare the OPEX and CAPEX cost distributions under the governance of Systems 1 on both C_2_H_5_OH and C_2_H_4_ to show key differences in cost distribution and corresponding MSPs with respect to operational scale. For instance, under a 1 TPD production scale, the MSPs of C_2_H_5_OH and C_2_H_4_ increase by 13.7 and 27.1%, respectively, compared to those at 100 TPD which is largely due to the non-linear scaling of CAPEX requirements with respect to scale – although electrolyzer-dependent CAPEX is considered linearly dependent on production scale. A summary scatter plot is presented in Fig. 6f for technology-product couples with a positive cumulative present value at the end of the 20 year plant lifetime assumed in the TEA NPV model. Technology-product dependent CPV *versus* operating time plots are presented in Fig. S2.† Therein, the two scenarios (considering or not considering byproduct revenue streams) are presented for each System. On average, both amine- and (bi)carbonate-mediated eRCC approaches require a longer payback period, or breakeven point, compared to the conventional route (System 1) or the emerging direct ACC (System 4). And this is not necessarily attributed to poor cell performance of liquid-based eRCC, but rather an apparent intrinsic disadvantage of requiring liquid-product separation and regeneration of the capture media.

## 6 Perspectives and recommendations

The evolving field of electrochemical reactive capture and conversion of CO_2_ (eRCC), namely amine- and (bi)carbonate-mediated eRCC, has witnessed considerable expansion due to the holistic system advantages over conventional electrochemical CO_2_ reduction. We have identified several component-level bottlenecks in eRCC – from transport limitations to constraints on the allowable target reaction products. Notwithstanding, the identification and explicit understanding of such challenges is a key step in circumventing the predicament. Furthermore, we have formulated and presented NPV-based TEA model results for 5 key products using contemporary cell performance results under the governance of conventional, eRCC, and emerging direct ACC. Our findings highlight several key areas of limitations particularly for eRCC technologies that target liquid-based products (alcohols, carboxylic acids, *etc.*) and the enormous OPEX separation costs from distillation units to separate said liquid-products from the reactive capture liquid solution. We briefly outline the primary bottlenecks for the eRCC systems investigated from a cell- and systems-level perspective.

For amine-mediated eRCC, challenges and overarching perspectives include:

• Limitations of C_2+_ products due to chemical and electrochemical instability of Cu-based electrocatalysts in conventional amines. It is suggested that testing of existing non-Cu based catalysts targeting C_2+_ products should be performed. Further, advances in computational density functional theory (DFT) and *in situ* material characterization techniques guide the rational design of novel materials that are electroactive, selective, and durable under amine-based electrolytes.

• Approximately 3 times higher viscosity of amine electrolytes is registered compared to concentrated conventional bicarbonate or hydroxides. This translates to mass transfer limitations for the reactive species (CO_2_ or amine–CO_2_ adduct) and is expected to curb current densities from reaching industrially relevant ranges. The investigation of temperature effects on amine-mediated eRCC is recommended. However, to prevent low CO_2_ adsorption capacities at higher temperatures, it is suggested that electrolyte engineering with respect to amine–CO_2_ binding affinities, p*K*_a_ values, steric effects of the amine, and the reactivity of the amine–CO_2_ adduct are to be developed further prior to the continued use of conventional amines for electrolysis.

• Although eRCC technologies are designed with the wide assumption that upstream saturation of CO_2_ in the capture solution will be achieved from a point-source emission, this is far from what has been investigated. To the best of our knowledge, pure CO_2_ or dry ice is used to saturate the capture solutions prior to electrochemical testing. This directly omits the presence of emission impurities (O_2_, SO_*x*_, NO_*x*_) which have been found to have critical effects on the catalytic performance. Therefore, strategies towards impurity agnostic amine-mediated eRCC should be developed.

• TEA from NPV models highlighted the criticality of FE and electroactivity (through low voltage requirements at a given current density) as primary cell-level factors for all products, irrespective of the eCO_2_RR technology employed. This translates to a feasibility window for amine-mediated eRCC to be industrially relevant given that (1) catalyst and electrolyzer components' stability is ensured and (2) only gaseous products or potentially high vapor pressure products are targeted if operating under higher temperatures. Liquid product(s) separation from the liquid amine electrolyte makes the process irreversibly non-feasible regardless of production scale.

For (bi)carbonate-mediated eRCC, some of the above-mentioned points are applicable. It's crucial to reiterate that point-source emissions, which ideally supply CO_2_ to bifunctional capture liquids and electrolytes (such as bicarbonate, amine, or ionic liquids), generally contain low CO_2_ concentrations. Achieving the conventional 3.0 M bicarbonate electrolyte directly from flue gas conversion into hydroxide is improbable without significant impurity inclusion. This is because, with the same hydroxide capture solution, it would take nearly ten times longer to reach equivalent bicarbonate concentrations from a 5 mol% CO_2_ feed compared to a 50 mol% feed, increasing the likelihood of impurities accumulating during the extended capture process. Some additives, such as DTAB, have been investigated to alleviate impurity effects on (bi)carbonate-mediated eRCC to CO with some success. However, their stability at different applied voltages, their effects on cell stability, and their effects on other catalysts targeting other products have not been studied, and therefore, the criticality for demonstrating a continuous impurity agnostic operation wherein the generation of the saturated (bi)carbonate electrolyte considers an impurity-ridden emission point-source. Also, the regeneration and separation of the (bi)carbonate electrolyte and liquid product(s), respectively, would result in nonsensical distillation requirements (OPEX and CAPEX) that would render the process non-feasible. Nevertheless, other challenges and perspective points that are relevant to (bi)carbonate-mediated eRCC include:

• Ensuring high CO_2_ reduction electroactivity is to ensure a non-transport limiting rate of *in situ* generated CO_2_ from (bi)carbonate. The rational control of GDE design parameters including, but not limited to, the distance between the catalyst and CEM side of the BPM, ionomer type and loading around the catalyst, and rate of proton flux from the CEM side of the BPM, amongst others, can lower transport limitations of CO_2_ in reaching the catalytic interface for reduction.

• It is advisable to examine temperature effects under extended operating conditions, as higher temperatures are anticipated to enhance current densities by accelerating the dissociation of bicarbonate into CO_2_. However, this improvement may come at the expense of cell component longevity, such as the potential degradation of interposers.

• High voltage requirements hinder the competitiveness of (bi)carbonate-eRCC for some target products, especially those with a higher number of electron transfer steps. This is largely due to the use of commercial BPMs that operate under reverse bias without the presence of WDCs. Using WDCs can substantially cut the operational costs and ensure a controllable flux of protons to the catholyte for *in situ* generation of CO_2_ without negatively contributing to undesired HER.

Since ACC is an emerging field, few works have been reported to date. However, guided by the above-mentioned perspective points for amine- and (bi)carbonate-mediated eRCC, the ACC approach is expected to circumvent many of the intrinsic challenges associated with them. These mainly include transport limitation issues of the reactive species, upstream capture solution preparation time and costs, and the regeneration and separation of capture electrolyte and liquid (by)products, respectively. Instead, the key recommendation points for work on ACC include:

• The need to test under varying degrees and types of impurities and relative humidity, and not pure CO_2_ streams – which beats the purpose of ACC.

• Identifying the potential zone of resistance in hybrid GDEs used in ACC, for instance, between the MMM pre-concentration layer and the catalytic layer. The nascence of this field allows for a myriad of hybrid GDE configurations, both using different material-group combinations and architectural arrangements.

• It is recommended to use CO_2_ pre-concentration materials that have no intrinsic electroactivity towards CO_2_ (or water) reduction. HKUST-1, which has been demonstrated as a MOF-based pre-concentration material in more than one report thus far, can reduce CO_2_ to CO, C_2_H_4_, and CH_4_. Therefore, any catalytic material downstream of it in the hybrid GDE will have its performance tainted by the electroactive pre-concentration layer.

• Both computational and advanced characterization techniques are recommended to be used synergistically towards the unravelling of potentially new interfacial phenomena in hybrid GDEs. This can guide the rational design of highly selective and kinetically facile solid sorbents in congruence with novel catalytic structures above or in a matrix with the pre-concentration sorbents.

It is perhaps central to realize that technoeconomic analyses are often conducted under idealized conditions, presenting best-case scenarios that focus on the idealistic potential economic benefits of emerging technologies. However, the real-world efficacy and applicability of these analyses are governed by factors that are frequently overlooked in current studies. Two key areas that are severely understudied in the context of electrochemical technologies in general, and electrochemical CO_2_ reduction in specific, are the long-term stability of these systems under industrially relevant current densities and their scalability for large-scale deployment. The performance and durability of electrolyzer systems over thousands of operational hours at high current densities are critical for assessing their viability beyond laboratory conditions. Similarly, the scalability of these systems to meet the demands of industrial applications remains a major challenge. The transition from small-scale single-cell (1–25 cm^2^) prototypes to large-scale multi-cellular stack systems (0.01–1 m^2^ cells) often involves unforeseen complexities primarily pertaining to deterioration in catalytic performance,^158^ and inhomogeneous performance distribution across scaled-up GDEs due to a plethora of identified reasons (*i.e.*, feed gas transport limitations downstream from inlet ports, varying compression stresses and Ohmic losses across larger GDEs).^159–161^ While ensuring a linear scale-up of performance metrics (selectivity, activity, conversion efficiency, and cathodic efficiency) is crucial, it has not yet been consistently achieved. Addressing these challenges individually requires a multidisciplinary framework and the transference of knowledge between the different eCO_2_RR technologies is not guaranteed *per se* due to differences in their respective governing chemistries. For a more nuanced discussion of challenges and advances in scalable eCO_2_RR under the conventional SCCC system, we refer the reader to the recent perspective work by Sun *et al.*^162^

Welcomed attempts at showcasing operational stability on the thousands-of-hours scale were only successfully demonstrated in a handful of studies.^152,163–166^ Further, this was toward the production of C_1_ products (CO and HCOOH) – arguably the simplest of all eCO_2_R target products in terms of electrochemistry and engineering management. This was also under the conventional SCCC design, with idealistic and well-controlled systems where no impurities were allowed to diffuse or permeate into feed reagents. Currently, the long-term effects of impurities on electrocatalytic stability are not well understood and should not be assumed to be universally applicable across different eCO_2_RR technologies, as these technologies involve distinct cell chemistry. Briefly, a primary reason behind durability issues in SCCC has been known to be the increased hydrophilicity of the GDE under operational conditions, which causes flooding and salt precipitation on the cathode side.^167,168^ To that end, a large portion of recent literature has investigated strategies that ensure sustained hydrophobicity during operation.^169^ Worthy of note is the very recent work by Chen *et al.* which employed highly hydrophobic conductive ionomers in place of conventional Nafion™.^170^ Therein, they demonstrated a degree of control over transport limitations through modulating the ratio of CO_2_/H_2_O within the surface microenvironment which enhanced C_2+_ activities and selectivity, as well as durability at ultra-high current densities. The aforementioned work exemplifies potential quantum leaps in cell performance enhancements. Notwithstanding, it is essential to bridge the gap between theoretical technoeconomic projections and the practical implementation of electrolyzer technologies at the industrial level through much more dedicated work that confronts stability and scalability.

All in all, the development of component level aspects of eRCC is expected to further drop the MSP of certain products (*i.e.*, C_2_H_4_) with a high market size below their corresponding average market price over the coming years. This is largely due to the elimination of carbonate crossover, and therefore recycle requirements at the anolyte effluent gas stream, which was shown to considerably add to levelized production costs.^157^ Further, innovative anolyte/catholyte schemes and configurations coupled with novel electrolyzer cell designs may circumvent the critical challenge of eRCC technologies with liquid products by performing the separation *in situ*, given that typical alcohols and carboxylic acids have a higher vapor pressure than conventional reactive capture agents (30% MEA and 3 M K_2_CO_3_). Moreover, for eRCC technology to be adaptable with their stated use-case of selectively enriching the capture agent with CO_2_ from flue gas for subsequent reduction, the adsorption selectivity of CO_2_ in said capture solutions needs to be improved. In the same vein, the agnosticism of the eRCC catalysts, and hybrid GDEs in the emerging direct ACC technology, to reactive impurities (SO_*X*_, NO_*X*_, O_2_) that are likely to be entailing from upstream flue gas needs to be further studied and ensured. Lastly, since eRCC technologies are still considered to be in their early years of development, it is understood that most studies undertaken tend to use conventional catalysts with little novelty. As more computational models and *in situ* studies emerge, clearer distinctions will be established between the microkinetic effects of CO_2_ reactive species, catalytic microenvironments during reaction, and impurity effects, and novel materials are expected to be developed which would be particularly suited towards eRCC.

In summary, while SCCC technologies are the most mature approach within eCO_2_RR, benefiting from high-TRL carbon capture methods and advancements in gas diffusion electrodes, their overall TRL remains limited by the electrochemical conversion step. Key products like CO and formic acid have achieved TRLs of 5–6 and 3–5, respectively, whereas most other targets, including tandem CO_2_ to CO to C_2_H_4_, rank at TRL 4 or lower.^35^ Emerging integrated ACC routes discussed in this perspective generally fall between TRLs of 3–4 due to unresolved challenges in selectivity, scalability, stability, and agnosticism toward feed impurities. Similarly, eRCC approaches are at a nascent stage, with TRLs likely being equal to or lower than those of SCCC for comparable products, underscoring the need for significant advancements to transition these technologies toward commercial viability.

## Data availability

Economic and financial model assumptions and NPV technoeconomic model formulation have been detailed in the ESI.†

## Author contributions

AB was the main contributor to the writing of the manuscript, whereas YL was the main contributor to its revision. Both authors actively participated in the scientific discussions that shaped the content of this perspective.

## Conflicts of interest

The authors have no competing interests to declare.

## Supplementary Material

SC-016-D4SC06642A-s001
