# Supplementary material for: A critical appraisal of advances in integrated CO2 capture and electrochemical conversion
Source: Chem Sci. 2025 Jan 24;16(6):2483–513. doi: 10.1039/d4sc06642a (PMC11758242; doi:10.1039/d4sc06642a)
Supplement: SC-016-D4SC06642A-s001 [file SC-016-D4SC06642A-s001.pdf]

Supplementary Information (SI) for Chemical Science.
This journal is © The Royal Society of Chemistry 2025

Supplemental Information

A Critical Appraisal of Advances in Integrated CO_2 Capture and Electrochemical Conversion

Ahmed Badreldin[1] and Ying Li[1,2]*

[1]J. Mike Walker '66 Department of Mechanical Engineering, Texas A&M University, College Station, TX 77843, USA

[2]Artie McFerrin Department of Chemical Engineering, Texas A&M University, College Station, TX 77843, USA

*Corresponding author; email: yingli@tamu.edu

Table S1. Technology-product dependent electrochemical CO2 reduction cell performance from surveyed contemporary literature.

	System 1	System 2	System 3	System 4
	Conventional Sequential Capture, Compression, Conversion	Amine-Mediated eRCC	(Bi)Carbonate-Mediated eRCC	Direct Conversion (ACC)
**CO2 to CO**				
Cell Voltage (V)	3	2.5	3	2.3
FE (%)	95	72	95	96
Current Density (mA cm^{-2})	200	50	100	120
SPCE (%)	40	80	40	60
Reference	Gang et al.[1]	Lee et al.[2]	Tester	Liu et al.[3]
**CO2 to HCOOH**				
Cell Voltage (V)	3	---	4	2.6
FE (%)	96	---	62	90
Current Density (mA cm^{-2})	260	---	100	80
SPCE (%)	40	---	40	60
Reference	Jia et al.[4]	---	Li et al.[5]	Zhao et al.[6]
**CO2 to CH3OH**				
Cell Voltage (V)	3.3	---	4	3.5
FE (%)	77.6	---	50	50
Current Density (mA cm^{-2})	41.5	---	100	100
SPCE (%)	50	---	60	60
Reference	Yang et al.[7]	---	Tester	Tester
**CO2 to C2H5OH**				
Cell Voltage (V)	3.5	---	4	3.5
FE (%)	52	---	30	30
Current Density (mA cm^{-2})	200	---	100	100
SPCE (%)	40	---	60	60
Reference	Gu et al.[8]	---	Tester	Tester
**CO2 to C2H4**				
Cell Voltage (V)	2.5	---	3.82	4.2
FE (%)	70	---	40	51
Current Density (mA cm^{-2})	200	---	300	525
SPCE (%)	40	---	35	60
Reference	Choi et al.[9]	---	Xie et al.[10]	Nam et al.[11]

Table S2. Process assumptions for CO2 Electrolyzer Model .

Parameter	System 1	System 2	System 3	System 4
	Conventional Sequential Capture, Compression, Conversion	Amine-Mediated eRCC	(Bi)Carbonate-Mediated eRCC	Direct Conversion (ACC)
Production Rate (ton day^{-1})	100			
Plant Lifetime (Years)	20			
Operating Days Per Year (day year^{-1})	350			
LCOE (USD kWh^{-1})	0.03			
Electrolyzer Cost Based on DOE H2A (USD m^{-2})[12]	919.669			
Electrolyzer Maintenance Cost wrt Capital (%)	2.5			
Capture Media	30% MEA	30% MEA	6.0 M KOH	Solid GDE
Capture Media Cost (USD ton^{-1})[13]	2454	2454	500	---
Absorption Capacity (mol$_{CO2}$ molsolvent^{-1})[14]	0.45			---
Capture Efficiency (%)	90			
Capture Capital Cost (USD kg^{-1})	0.000049	---	---	---
PSA Reference Cost (USD)	1989043			
PSA Scaling Factor	0.7			
PSA Reference Capacity (m^3 hr^{-1})	1000			
PSA Electricity Operating Cost (kWh m^{-3})	0.25			
Compressor Reference Cost (0.82 scaling factor)	5840			
Distillation Reference Capacity (kg h^{-1})	77469			
Distillation Reference Cost (USD)	15077002			
Distillation Reference Operating Cost (USD day^{-1})	69455			
Carbonate Formation (%)	30	---	---	---
Labor Cost wrt to Total CAPEX (%)	5			
Electrolyzer Replacement Cost	** 15% of Electrolyzer Capital Cost. Replaced every 5 years.			
Water Cost (USD gal^{-1})	0.0054			

Table S3. Financial assumptions for NPV Model.

Parameter	All Systems
Income Tax (%)	38.9
Discount Rate (%)	10
Market Price of Hydrogen (USD kg^{-1})	1.9
Market Price of Oxygen (USD kg^{-1})	0.4
Market Price of CO (USD kg^{-1})	0.6
Market Price of HCOOH (USD kg^{-1})	0.88
Market Price of CH3OH (USD kg^{-1})	0.62
Market Price of C2H5OH (USD kg^{-1})	0.77
Market Price of C2H4 (USD kg^{-1})	1.02

Supplementary Note 1: Sample Calculation of OPEX and CAPEX Parameters for CO_2 electrochemical reduction through the conventional System 1 towards the 2 electron-transfer HCOOH production. The PFD shown in Figure 1a is relevant to this exercise.

Flowrates of Streams – System 1 (HCOOH)

Based on a production rate of 100,000 kg day^{-1} of HCOOH at the cathode, the partial current density needed for the 2-electron reaction is:

$$Partial\ Current\ Needed: \frac{100{,}000\ kg}{day} x \frac{day}{86{,}400s} x \frac{1000\ g}{kg} x \frac{mol}{46.03\ g} x\ 2e^{-} x \frac{96{,}485\ C}{mol} = 4{,}852{,}160\ A$$

The total current density can be obtained by factoring for the 96% HCOOH FE noted in Table S1 for HCOOH under the governance of System 1:

$$Total\ Current\ Needed: \frac{4{,}852{,}160\ A}{0.96} = 5{,}054{,}333\ A$$

The total electrolyzer area (m2) needed can then be obtained by dividing the total current by the cell-specific current density noted in Table S1:

$$Electrolyzer\ Area\ Needed: \frac{5{,}054{,}333\ A}{\frac{0.260\ A}{cm^2}} x \frac{m^2}{10^4 cm^2} = 1{,}944\ m^2$$

Power requirement for the aforementioned electrolyzer is obtained by Power = Voltage x Current:

$$Required\ Power: 3\ V\ x\ 5{,}054{,}333\ A\ x\ \frac{W}{10^6 MW} = 15.16\ MW$$

The needed flowrate of CO_2 for its conversion to HCOOH is:

$$CO2\ Flowrated\ Needed\ for\ Reaction: \left[\frac{4{,}852{,}160\ A\ x\ \frac{86{,}400\ s}{day}\ x\ \frac{0.044\ kg\ CO2}{mol}}{\frac{2e^{-}}{mol\ CO2} x \frac{96{,}485\ C}{mol}}\right] = 95{,}590\ \frac{kg}{day}$$

Due to the lost SPCE for HCOOH production under System 1 being 40%, the actual CO_2 mass flowrate that needs to be fed to the cathode is:

$$CO2\ Flowrated\ Needed\ for\ Cell: \left[\frac{95{,}590\ \frac{kg}{day}}{0.4}\right] = 238{,}975\frac{kg}{day}$$

The outlet unreacted CO_2 is then needed in volumetric flowrate units for downstream sizing of separation equipment (i.e., PSA). Unreacted CO_2 is due to non-idea SPCE at the cathode side:

$$CO2\ Volumetric\ Flowrated\ at\ Outlet\ of\ Cathode: \left[\frac{238{,}975\frac{kg}{day}\ x\ (1-0.4)}{\frac{1.98\ kg\ CO2}{m^3}x\frac{24\ hr}{day}}\right] = 3{,}017\ \frac{m^3}{hr}$$

At the cathode side, the produced HCOOH will be modeled to be in liquid phase. Again, to obtain the volumetric flowrate of HCOOH produced (for downstream sizing of the distillation unit), the following can used assuming a density of 1,220 kg m^{-3} for HCOOH. It is worth noting that for all liquid products, the process is modeled and sized under the assumption of a 30 wt.% content of the target product in a water balance.

$$HCOOH\ Flowrate\ out\ of\ Cathode: \left[\frac{4{,}852{,}160\ A}{\frac{2e^-}{mol\ CO2}x\frac{96{,}485\ C}{mol}}\right] x \left[\frac{\frac{0.04603\ kg}{mol}}{\frac{1{,}220\ kg}{m^3}}\right] x\frac{3{,}600s}{hr} = 3.415\ \frac{m^3}{hr}$$

For all modeled technology-product couples, we assume that H2 is the sole cathodic byproduct at FE < 100 % for the target product. In this case, since the FE for HCOOH is 96%, a corresponding FE of 4% is for H_2. To size downstream gas separation units, the total gas flowrate from both the cathode (and anode) needs to be considered. A density of 0.0899 kg m^{-3} is used for H2 gas under process conditions.

$$H2\ Flowrate\ out\ of\ Cathode: \left[\frac{5{,}054{,}333\ A\ x\ (1-0.96)}{\frac{2e^-}{mol\ CO2}x\frac{96{,}485\ C}{mol}}\right] x \left[\frac{\frac{0.002\ kg}{mol}}{\frac{0.0899\ kg}{m^3}}\right] x\frac{3{,}600s}{hr} = 83.909\ \frac{m^3}{hr}$$

The total gas flowrate out of the cathode comprising of unreacted CO_2 and evolved H_2 is therefore simply the addition of their volumetric flowrates (83.909 + 3,017) m^3 hr^{-1} = 3,101.3 m^3 hr^{-1}.

The water flowrate needed for the 2-electron transfer cathodic HER is:

$$Needed\ H2O\ for\ H2: \left[\frac{5{,}054{,}333\ A\ x\ (1-0.96)}{\frac{2e^-}{mol\ CO2}x\frac{96{,}485\ C}{mol}}\right] x\frac{0.018\ kg}{mol}x\ 0.2642\frac{gal}{kg}x\frac{86{,}400s}{day} = 421.51\ \frac{gal}{day}$$

The water flowrate needed for dilution of the produced liquid product is simply based on achieving 30 wt.% of the product as it exits the electrolyzer. In doing so, this allows a higher concentration of HCOOH to reach the distillation, making separation more facile:

$$Needed\ H2O\ for\ HCOOH\ mix: 3.415\ \frac{m^3}{hr}x\frac{0.7}{0.3}x\ 264.2\frac{gal}{m^3} = 2{,}105.42\frac{gal}{day}$$

At the anode side, two primary things occur under System 1 – irrespective of the target cathodic product. The carbonate which formed in-situ from CO2 acid-base chemistry with the catholyte is allowed to pass through the AEM (anion exchange membrane) from the cathode to the anode side. At the anode, carbonate oxidizes back to CO2. Simultaneously, water oxidation at the anode is modeled, which produces O2 through the conventional 4-electron transfer anodic process (oxygen evolution reaction). Therefore, at the anode for System 1, both CO2 and O2 are effluent gases that need to be separated. CO2 is recycled to the cathode and O2 can be sold if purified to market standard. The density of O2 is taken as 1.429 kg m^{-3}.

$$CO2\ Volumetric\ Flowrated\ at\ Outlet\ of\ Anode: \left[\frac{238{,}975\,\frac{kg}{day}\ x\ (1-0.3)}{\frac{1.98\ kg\ CO2}{m^3}\ x\ \frac{24\ hr}{day}}\right] = 1{,}508.68\ \frac{m^3}{hr}$$

$$O2\ Volumetric\ Flowrated\ at\ Anode: \left[\frac{4{,}852{,}160\ A}{\frac{4e^-}{mol\ CO2}\ x\ \frac{96{,}485\ C}{mol}}\right] x \left[\frac{\frac{0.032\ kg}{mol}}{\frac{1.429\ kg}{m^3}}\right] x\ \frac{3{,}600s}{hr} = 1{,}056\,\frac{m^3}{hr}$$

The total effluent anodic gas flowrate is therefore (1,056 + 1,508.68) $m^3\ hr^{-1}$ ~ 2,565 $m^3\ hr^{-1}$.

The water needed at the anode for the anodic OER is:

$$Needed\ H2O\ for\ O2: \left[\frac{5{,}054{,}333\ A}{\frac{4e^-}{mol\ CO2}\ x\ \frac{96{,}485\ C}{mol}}\right] x\ \frac{0.018\ kg}{mol}\ x\ 0.2642\frac{gal}{kg}\ x\ \frac{86{,}400s}{day} = 5{,}268.9\ \frac{gal}{day}$$

This is mentioned here for reference only since this exercise is on System 1, but for liquid-based eRCC Systems (i.e., System 3 for HCOOH), if cell parameters from Table S1 are used, an additional accounting of reactive capture electrolyte would need to be done. For reference, it would come to be equal to 2,158,918 kg day^{-1}. Not only is it by far the largest liquid-based flowrate of the system (and therefore the raw cost of the electrolyte is high), but more importantly CAPEX and OPEX sizing equations for distillation units are based on non-linearly sized from flowrate. This reflects the huge costs associated with liquid-mediated eRCC Systems 2 and 3 for target products which are liquids (i.e., HCOOH, CH3OH, C2H5OH). This is discussed more in the paper.

CAPEX Calculations – System 1 (HCOOH)

CAPEX Calculations (as per Figure 1a for System 1) will involve the electrolyzer cost, separation units for gas (PSA) and liquid (distillation), the capital cost for the compressors which take the purified CO_2 from the upstream strippers to the electrolyzer, the amine-based absorption and stripping towers, and the Balance of Plant (BoP). The electrolyzer sizing is based on the DOE H2A analysis for central grid electrolysis which corresponds to a stack cost of 250.25 USD kW^{-1}. Therein, the electrolyzer used in the H2A model requires 1.75 V to sustain 175 mA cm^{-2} of current density and the installation factor is 1.2. Therefore, to get the normalized cost per unit area for the electrolyzer the following calculation is performed:

$$Reference\ Electrolyzer\ Cost\,\frac{1.2\ x\ 0.175A}{cm^2}\ x\ \frac{250.25\ USD}{kW}\ x\,\frac{10^4 cm^2 x\ kW}{m^2\ x\ 10^3 W}\ x\ 1.75\ V = 919.67\ \frac{USD}{m^2}$$

Knowing that 1,944 m^2 of electrolyzer area is needed, the Electrolyzer CAPEX is therefore:

$$Electrolyzer\ Cost: 919.67\ \frac{USD}{m^2}\ x\ 1{,}944\ m^2 = 1{,}787{,}812\ USD$$

Following the H2A Model, the BoP for the electrolyzer is 35% of the total cost, with the balance being for the stack. Therefore, the BoP capital cost is 1,787,812 x (0.35/0.65) = 962,668 USD.

With an upstream levelized capture capital cost of 0.000049 USD kg^{-1}, the CO_2 capture capital cost is simply the needed CO2 flowrate for the cell multiplied by the levelized cost:

$$CO2\ Capture\ Cost: 238{,}975 \frac{kg}{day}\ x\ 0.000049\ \frac{USD}{kg}\ x\ 350\ \frac{days}{year}\ x\ 20\ years = 81{,}968\ USD$$

As alluded to, since this is a liquid-target product example, only one PSA will be needed at the cathode to separate the unreacted CO2 from the byproduct H2, and one PSA will be needed at the anode gas effluent to separate CO2 (from crossed over carbonate) from the O2 evolved at the anode. It is worth noting that when the target product is gas (i.e., CO), two PSAs will be needed at the cathode in series. The first will separate the CO2 from the H2 and CO, and the second will separate the CO from H2. Notwithstanding, the CAPEX for the PSAs used in this example are as follows:

$$PSA\ Capital\ Cost: 1{,}989{,}043\ USD\ x \left[\left(\frac{\frac{3{,}101.3\ m^3}{hr}}{\frac{1000\ m^3}{hr}}\right)^{0.7} + \left(\frac{\frac{2{,}565\ m^3}{hr}}{\frac{1000\ m^3}{hr}}\right)^{0.7}\right] = 8237970\ USD\text{s}$$

For obtaining the capital cost needed for the compressors, the power needed (assuming 72% compressor efficiency) is obtained assuming the stream is pure CO2 at a flowrate equivalent to the inlet CO2 flowrate needed for the cell. For simplicity, and assuming the upstream CO2 purification step is very close to the electrolyzer such that no significant pressure drops during transfer will be noted, a design discharge pressure of 0.1 barg is used to obtain the needed work. For example, under the CO2 flowrate of 238,975 kg day^{-1} (equivalent to 5028.9 m^3 hr^{-1}) the needed work is 18.7 kW. Using the Douglas relation, the CAPEX for the compressor is:

$$Compressor\ Capital\ Cost: 5{,}840\ USD\ x\ (18.7\ kW)^{0.82} = 64{,}465\ USD$$

However, for comparison if the target discharge pressure needed was for longer distances and to consider pressure drops along the pipeline – assuming discharge pressure of 2 barg – the work needed to compress the same fluid would be 243.7 kW. This would bring the capital cost of the compressor to 529,218 USD. Notwithstanding, for all System 1 products in this work, a 0.1 barg discharge pressure was assumed.

Since this product is a liquid, we need distillation to purify the product stream. This is calculated by scaling the reference cost of the total liquid flowrate. The division by 0.3 is to account for the fact that the process was modeled with 30 wt.% HCOOH and therefore the remaining 70% water needs to be accounted for when sizing the distillation:

$$Distillation\ Capital\ Cost: 1{,}5077{,}002\ USD\ x \left(\frac{\frac{100{,}000\ kg}{day}\ x\ \frac{day}{24\ hr}\ x\ \frac{1}{0.3}}{\frac{77{,}469\ kg}{hr}}\right)^{0.7} = 4{,}526{,}814\ USD$$

Taking this into account, the total CAPEX comes to 15,579,741 USD.

OPEX Calculations – System 1 (HCOOH)

The electricity cost of the electrolyzer operation is calculated with a LCOE of 0.03 USD kWh^{-1} as noted in Table S2.

$$Electrolyzer\ Operating\ Cost: 15.16\ MW\ x\ \frac{1{,}000\ kW}{MW}\ x\ \frac{0.03\ USD}{kWh}\ x\ \frac{24\ hrs}{day} = 10917\frac{USD}{day}$$

Maintenance cost of the electrolyzer is based on H2A model, wherein it is assumed to be 2.5% of the electrolyzer capital cost per year:

$$Electrolyzer\ Maintenance\ Cost: 1{,}787{,}812\ \frac{USD\ x\ year}{350\ days}\ x\ 0.025 = 128\ \frac{USD}{day}$$

The replacement cost of cell components is taken as 15% of the electrolyzer capital cost levelized per day and replacement is conservatively assumed every 5 years:

$$Labor\ Cost: 1{,}787{,}812\ \frac{USD\ x\ year}{350\ days}\ x\ \frac{0.15}{5\ year} = 153\frac{USD}{day}$$

The PSA operating cost is based on the scaling the gas flowrates (both anode and cathode since it is a linear relation) with the scaling parameter noted in Table S2 (0.25 kWh m^{-3}) as follows:

$$PSAs\ Operating\ Cost: \frac{0.25\ kWh}{m^3}\ x\ \left(\frac{2565\ m^3}{hr} + \frac{3{,}101.3m^3}{hr}\right) x\frac{0.03\ USD}{kWh}\ x\frac{24\ hrs}{day} = 1020\frac{USD}{day}$$

Based on the known work of Rochelle et al.,[15] the cost information for CO2 scrubbing from MEA in Table S2 is obtained and modeled with 100% thermal efficiency. To that end, the upstream CO2 capture energy operating cost is calculated as follows:

$$CO2\ Capture\ Energy\ Required: 238{,}975\frac{kg}{day}\ x\frac{3.8\ GJ}{ton}\ x\ \frac{ton}{10^3 kg} = 908\ \ \frac{GJ}{day}$$

$$CO2\ Capture\ Energy\ Operating\ Cost: 908\frac{GJ}{day}\ x\frac{1\ MWh}{3.6\ GJ}\ x\frac{0.03\ USD}{kWh}x\ \frac{10^3 kWh}{MWh} = 7{,}568\frac{USD}{day}$$

Compression operating cost is then calculated based on 0.35 GJ ton^{-1} as follows:

$$CO2\ Compression\ Energy\ Required: 238{,}975\frac{kg}{day}\ x\frac{0.35\ GJ}{ton}\ x\ \frac{ton}{10^3 kg} = 84\ \ \frac{GJ}{day}$$

$$CO2\ Compression\ Energy\ Operating\ Cost: 84\frac{GJ}{day}\ x\frac{1\ MWh}{3.6\ GJ}\ x\frac{0.03\ USD}{kWh}x\ \frac{10^3 kWh}{MWh} = 697\frac{USD}{day}$$

Next, the upstream 30% MEA sorbent capture cost is calculated as:

$$Upstream\ Sorbent\ Operating\ Cost: 238{,}975\frac{kg}{day}\ x\ \frac{mol\ CO2}{0.044\ kg}\ x\ \frac{mol\ MEA}{(0.45\ mol\ CO2)\ x\ (0.9)}\ x$$

$$\frac{61.08\ x\ 10^{-6}\ ton\ MEA}{mol\ MEA}\ x\ \frac{2425\ USD}{ton\ MEA}\ x\ \frac{year}{20\ year\ x\ 350\ days} = 284\frac{USD}{day}$$

The distillation operating cost is then calculated based on the scaling parameters in Table S2:

$$Distillation\ Operating\ Cost: 69{,}455 \frac{USD}{day}\ x \left(\frac{\frac{100{,}000\ kg}{day}\ x\ \frac{day}{24\ hr}\ x\ \frac{1}{0.3}}{\frac{77{,}469\ kg}{hr}} \right) = 12{,}452\ \frac{USD}{day}$$

The water operating cost is calculated based on the cost of needed water flowrate for cathodic and anodic processes:

$$Water\ Operating\ Cost: 0.0054 \frac{USD}{gal}\ x\ \left(5{,}268.9\ \frac{gal}{day} + 421.51\ \frac{gal}{day} + 2{,}105.42 \frac{gal}{day}\right) = 42 \frac{USD}{day}$$

The labor cost is taken as 5% of the total CAPEX levelized per day:

$$Labor\ Cost: \frac{15{,}579{,}741\ USD\ x\ 0.05}{20\ year\ x\ 350\ \frac{days}{year}} = 111 \frac{USD}{day}$$

Therefore, the total OPEX is 33,372 USD day^{-1}.

TableS4. System-dependent OPEX breakdown for 100-ton day^{-1} CO production.

	System 1	System 2	System 3	System 4
Electrolyzer Electric	36%	54%	64%	52%
Electrolyzer Maintenance	1%	4%	2%	2%
PSA	6%	3%	5%	7%
Capture Energy	24%	0%	0%	0%
Compression	2%	0%	0%	0%
Sorbent	1%	1%	0%	0%
Water	0%	0%	0%	0%
Labor	17%	3%	1%	2%
Component Replacement	3%	5%	2%	2%
Capital Depreciation	7%	15%	9%	12%
Average Income Tax	3%	16%	18%	23%

TableS5. System-dependent OPEX breakdown for 100-ton day^{-1} HCOOH production.

	System 1	System 3	System 4
Electrolyzer Electric	30%	3%	37%
Electrolyzer Maintenance	0%	0%	2%
Separations	37%	96%	47%
Capture Energy	21%	0%	0%
Compression	2%	0%	0%
Sorbent	1%	0%	0%
Water	0%	0%	0%
Labor	0%	0%	0%
Component Replacement	0%	0%	2%
Capital Depreciation	6%	1%	9%
Average Income Tax	3%	0%	4%

TableS6. System-dependent OPEX breakdown for 100-ton day^{-1} CH_3OH production.

	**System 1**	**System 3**	**System 4**
Electrolyzer Electric	53%	13%	75%
Electrolyzer Maintenance	4%	0%	2%
Separations	12%	84%	10%
Capture Energy	7%	0%	0%
Compression	1%	0%	0%
Sorbent	0%	0%	0%
Water	0%	0%	0%
Labor	1%	0%	0%
Component Replacement	4%	0%	2%
Capital Depreciation	13%	1%	7%
Average Income Tax	5%	1%	3%

TableS7. System-dependent OPEX breakdown for 100-ton day^{-1} C_2H_5OH production.

	**System 1**	**System 3**	**System 4**
Electrolyzer Electric	73%	22%	80%
Electrolyzer Maintenance	1%	0%	2%
PSA	9%	74%	5%
Capture Energy	8%	0%	0%
Compression	1%	0%	0%
Sorbent	1%	0%	0%
Water	0%	0%	0%
Labor	0%	0%	0%
Component Replacement	1%	1%	3%
Capital Depreciation	5%	2%	7%
Average Income Tax	2%	1%	3%

TableS8. System-dependent OPEX breakdown for 100-ton day^{-1} C2H4 production.

	System 1	System 3	System 4
Electrolyzer Electric	68%	92%	95%
Electrolyzer Maintenance	1%	1%	0%
PSA	4%	1%	1%
Capture Energy	14%	0%	0%
Compression	1%	0%	0%
Sorbent	1%	0%	0%
Water	0%	0%	0%
Labor	0%	0%	0%
Component Replacement	1%	1%	0%
Capital Depreciation	7%	3%	2%
Average Income Tax	3%	1%	1%

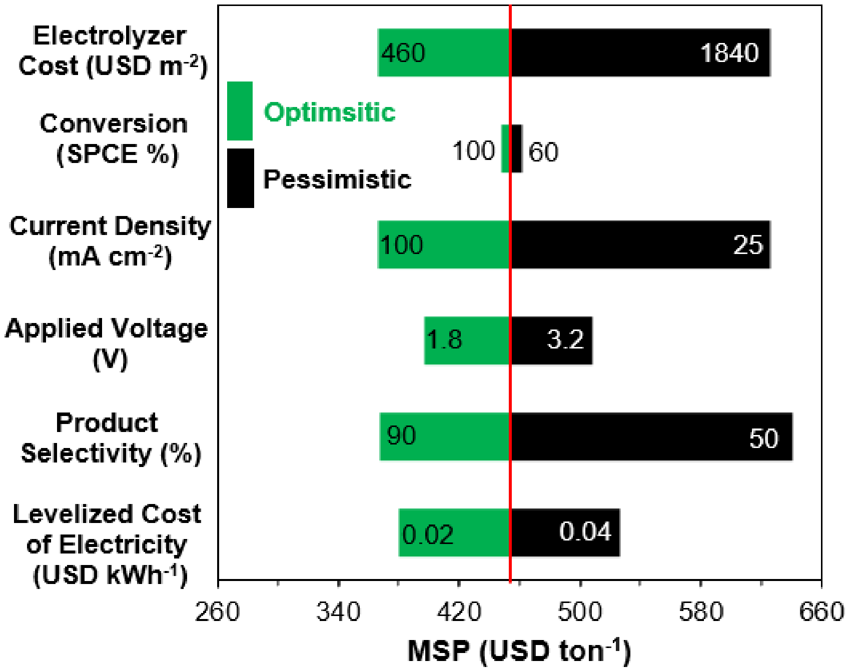

Figure S1. Sensitivity analysis of System 2 (amine-mediated eRCC) for CO Production.

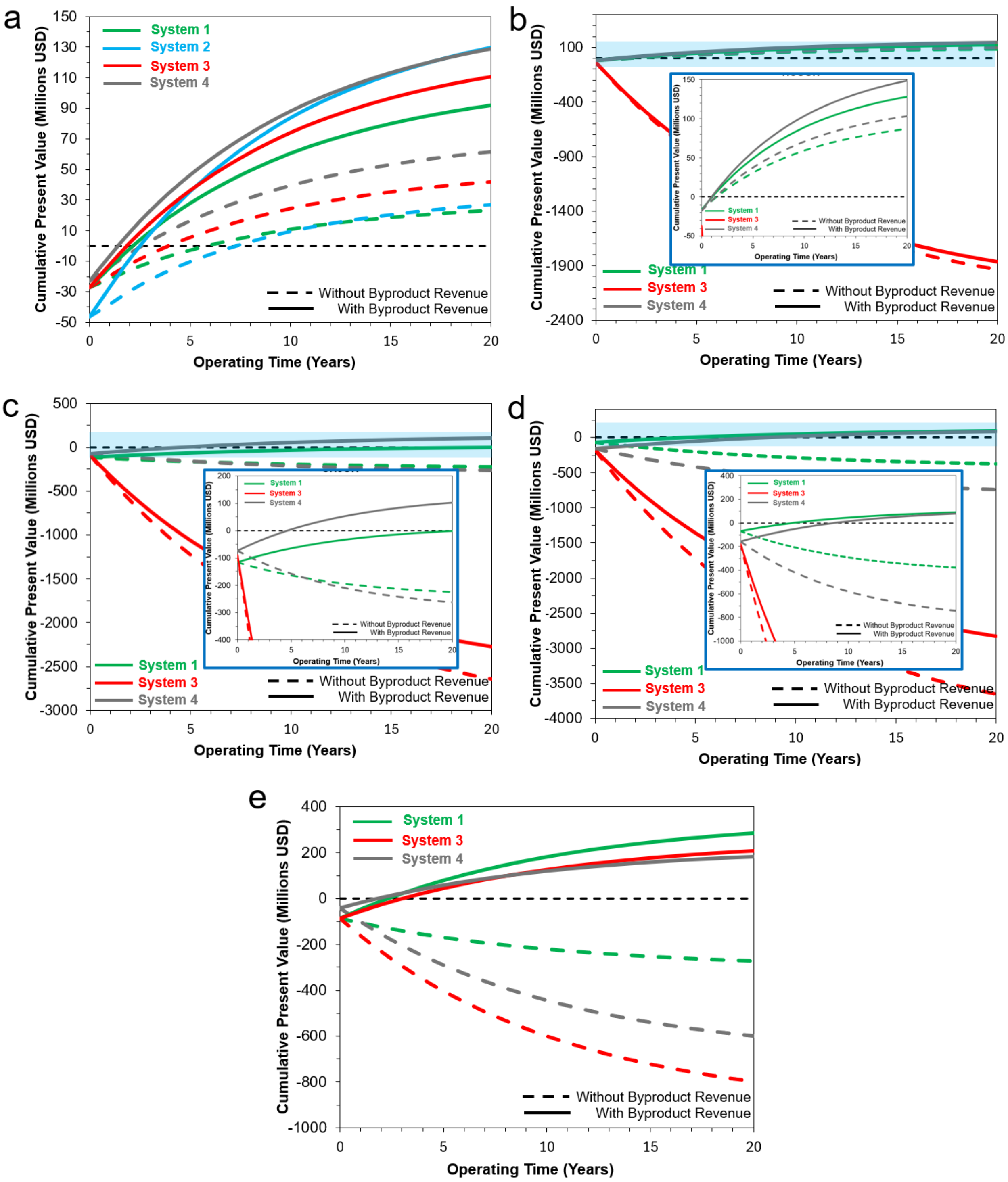

Figure S2. (a) Cumulative Present Value (CPV) vs. Years for CO, (b) HCOOH, (c) CH3OH, (d) C2H5OH, and (e) C2H4 based on the different technologies/systems and with and without consideration of accounting for revenue streams of byproducts H2 and O2.

References

(1) Gang, Y.; Li, B.; Fang, S.; Pellessier, J.; Fang, L.; Pan, F.; ... & Li, Y. Efficient electrochemical CO2 reduction to CO by metal and nitrogen co-doped carbon catalysts derived from pharmaceutical wastes adsorbed on commercial carbon nanotubes. *Chemical Engineering Journal* **2023**, *453*, 139712.

(2) Lee, G.; Li, Y. C.; Kim, J. Y.; Peng, T.; Nam, D. H.; Sedighian Rasouli, A.; ... & Sargent, E. H. Electrochemical upgrade of CO2 from amine capture solution. *Nature Energy* **2021**, *6* (1), 46-53.

(3) Liu, Y. Y.; Huang, J. R.; Zhu, H. L.; Liao, P. Q.; & Chen, X. M. Simultaneous Capture of CO2 Boosting Its Electroreduction in the Micropores of a Metal–organic Framework. *Angewandte Chemie* **2023**, *135* (52), e202311265.

(4) Jia, B.; Chen, Z.; Li, C.; Li, Z.; Zhou, X.; Wang, T.; ... & Zhang, B. Indium cyanamide for industrial-grade CO2 electroreduction to formic acid. *Journal of the American Chemical Society* **2023**, *145* (25), 14101-14111.

(5) Li, T.; Lees, E. W.; Zhang, Z.; & Berlinguette, C. P. Conversion of bicarbonate to formate in an electrochemical flow reactor. *ACS Energy Letters* **2020**, *5* (8), 2624-2630.

(6) Zhao, Z. H.; Huang, J. R.; Huang, D. S.; Zhu, H. L.; Liao, P. Q.; & Chen, X. M. Efficient Capture and Electroreduction of Dilute CO2 into Highly Pure and Concentrated Formic Acid Aqueous Solution. *Journal of the American Chemical Society* **2024**, *146* (20), 14349-14356.

(7) Yang, D.; Zhu, Q.; Chen, C.; Liu, H.; Liu, Z.; Zhao, Z.; ... & Han, B. Selective electroreduction of carbon dioxide to methanol on copper selenide nanocatalysts. *Nature communications* **2019**, *10* (1), 677.

(8) Gu, Z.; Shen, H.; Chen, Z.; Yang, Y.; Yang, C.; Ji, Y.; ... & Zheng, G. Efficient electrocatalytic CO2 reduction to C2+ alcohols at defect-site-rich Cu surface. *Joule* **2021**, *5* (2), 429-440.

(9) Choi, C.; Kwon, S.; Cheng, T.; Xu, M.; Tieu, P.; Lee, C.; ... & Huang, Y. Highly active and stable stepped Cu surface for enhanced electrochemical CO2 reduction to C2H4. *Nature catalysis* **2020**, *3* (10), 804-812.

(10) Xie, K.; Miao, R. K.; Ozden, A.; Liu, S.; Chen, Z.; Dinh, C. T.; ... & Sargent, E. H. Bipolar membrane electrolyzers enable high single-pass CO2 electroreduction to multicarbon products. *Nature communications* **2022**, *13* (1), 3609.

(11) Nam, D. H.; Shekhah, O.; Ozden, A.; McCallum, C.; Li, F.; Wang, X.; ... & Sargent, E. H. High-Rate and Selective CO2 Electrolysis to Ethylene via Metal–Organic-Framework-Augmented CO2 Availability. *Advanced Materials* **2022**, *34* (51), 2207088.

(12) Jouny, M.; Luc, W.; & Jiao, F. General techno-economic analysis of CO2 electrolysis systems. *Industrial & Engineering Chemistry Research* **2018**, *57* (6), 2165-2177.

(13) Davis, J.; & Rochelle, G. Thermal degradation of monoethanolamine at stripper conditions. *Energy Procedia* **2009**, *1* (1), 327-333.

(14) Chai, S. Y. W.; Ngu, L. H.; & How, B. S. Review of carbon capture absorbents for CO2 utilization. *Greenhouse Gases: Science and Technology* **2022**, *12* (3), 394-427.

(15) Rochelle, G. T. Amine scrubbing for CO2 capture. *Science* **2009**, *325* (5948), 1652-1654.
